# Network Geometry of Borsa Istanbul: Analyzing Sectoral Dynamics with Forman–Ricci Curvature

**DOI:** 10.3390/e27030271

**Published:** 2025-03-05

**Authors:** Ömer Akgüller, Mehmet Ali Balcı, Larissa Margareta Batrancea, Lucian Gaban

**Affiliations:** 1 Deparment of Mathematics, Faculty of Science, Muğla Sıtkı Koçman University, 48000 Muğla, Turkey; oakguller@mu.edu.tr; 2 Department of Business, Babeş-Bolyai University, 7 Horea Street, 400174 Cluj-Napoca, Romania; larissa.batrancea@ubbcluj.ro; 3 Faculty of Economics, “1 Decembrie 1918” University of Alba Iulia, 510009 Alba Iulia, Romania

**Keywords:** financial networks, Forman–Ricci curvature, mutual information, sectoral dynamics

## Abstract

This study investigates the dynamic interdependencies among key sectors of Borsa Istanbul—industrial, services, technology, banking, and electricity—using a novel network-geometric framework. Daily closure prices from 2022 to 2024 are transformed into logarithmic returns and analyzed via a sliding window approach. In each window, mutual information is computed to construct weighted networks that are filtered using Triangulated Maximally Filtered Graphs (TMFG) to isolate the most significant links. Forman–Ricci curvature is then calculated at the node level, and entropy measures over k-neighborhoods (k=1,2,3) capture the complexity of both local and global network structures. Cross-correlation, Granger causality, and transfer entropy analyses reveal that sector responses to macroeconomic shocks—such as inflation surges, interest rate hikes, and currency depreciation—vary considerably. The services sector emerges as a critical intermediary, transmitting shocks between the banking and both the industrial and technology sectors, while the electricity sector displays robust, stable interconnections. These findings demonstrate that curvature-based metrics capture nuanced network characteristics beyond traditional measures. Future work could incorporate high-frequency data to capture finer interactions and empirically compare curvature metrics with conventional indicators.

## 1. Introduction

The Turkish financial market has experienced substantial turbulence and transformation in recent years, shaped by a confluence of domestic policy shifts—often involving unconventional monetary measures—and global economic forces that have influenced capital flows and investor sentiment [1,2,3,4,5,6]. Within this dynamic landscape, the Borsa Istanbul (BIST) stands out as a focal point for both local and international investors, reflecting real-time adjustments to volatility in exchange rates, unpredictable inflationary pressures, and shifting credit conditions. This volatility is further compounded by Turkey’s unique economic structure, characterized by a significant reliance on external financing, a prominent role in the banking sector, and a diverse array of industries that are highly sensitive to both domestic and international economic indicators.

Despite the apparent significance of these factors, many established analytical frameworks rely primarily on linear correlation methods, which may oversimplify the complex interactions among assets. Traditional models, such as the Capital Asset Pricing Model or the Arbitrage Pricing Theory, often assume linear relationships and normal distributions of returns, assumptions that are frequently violated in emerging markets like Turkey [7,8,9,10]. These limitations can lead to inaccurate risk assessments and suboptimal investment strategies, particularly in environments marked by high volatility and structural economic changes [11,12,13,14]. This shortfall highlights a pressing need for more robust, nuanced tools capable of capturing the dense web of dependencies that characterize emerging markets, especially across multiple sectors that do not respond in isolation to macroeconomic triggers but rather exhibit intricate, interlinked reactions.

Motivated by these considerations, the present study adopts a multifaceted approach that integrates information theory, network science, and discrete geometry to examine four key BIST sectors: industrial, services, technology, and banking. By leveraging these advanced analytical techniques, we aim to uncover deeper insights into the structural and dynamic properties of sectoral interactions, providing a more granular understanding of market behavior under stress.

The initial step involves transforming raw stock prices into logarithmic returns—a common practice in financial modeling to stabilize variance and address potential non-stationarities. Logarithmic returns are preferred over simple returns as they facilitate additive properties over time and mitigate the impact of extreme values, thereby enhancing the robustness of subsequent analyses. We then employ mutual information, which surpasses simple correlation measures by detecting nonlinear dependencies across time-series data. Unlike Pearson correlation, mutual information can capture complex, nonlinear relationships, making it particularly suitable for uncovering hidden dependencies that linear methods might miss [15,16,17,18,19].

Drawing on the concept of Triangulated Maximally Filtered Graphs (TMFG), we reduce the dimensionality of these high-dimensional mutual information networks while retaining the most essential connections. TMFG is a powerful tool for filtering networks, as it preserves the most significant relationships based on a principled graph-theoretic approach, ensuring that the resulting structure is both sparse and informative [20,21,22]. This filtering process yields sparser and more interpretable graphs, setting the stage for Forman–Ricci curvature analysis. By assigning a curvature value to each node, we incorporate a geometric interpretation of network robustness: Curvature captures how “tight” or “loose” the immediate or extended neighborhood is, providing a measure of the structural role each firm occupies within the sector’s connectivity.

In constructing our network-geometric framework, we opted for TMFG over significantly simpler methodologies, such as a basic minimal spanning tree (MST) or the largest eigenvalue of the mutual information matrix, due to its superior ability to capture nuanced structural properties. While MSTs provide a straightforward approach to filtering networks, they are inherently limited by their restrictive tree-like structure, which severely constrains the diversity of neighborhoods needed to compute meaningful entropy measures across different scales [23,24]. Similarly, relying solely on the largest eigenvalue of the mutual information matrix would fail to encode the rich geometric insights that emerge from curvature-based analyses, as it reduces the network to a single scalar value, neglecting localized and hierarchical interdependencies [25,26]. TMFG, on the other hand, strikes an optimal balance by preserving both local clustering and broader connectivity patterns through its planar triangulation, enabling us to analyze entropies of curvatures across multiple neighborhood levels. This richer representation allows for a more comprehensive understanding of how local and global structures evolve under varying macroeconomic conditions. Although alternative methods like Planar Maximally Filtered Graph (PMFG) filtration could also offer detailed structural insights [27], they are computationally expensive and less scalable compared to TMFG, making the latter a more practical choice for dynamic financial network analysis.

Forman–Ricci curvature, rooted in discrete geometry, extends the classical notion of Ricci curvature from continuous manifolds to discrete networks [28,29,30]. It quantifies the curvature of edges and nodes by evaluating the balance between local clustering and the distribution of connections. Specifically, for each edge in the network, Forman–Ricci curvature considers the weights of the edge itself and the weights of adjacent edges, effectively capturing the interplay between a node and its immediate neighbors. This metric is particularly adept at identifying regions of the network that are either highly interconnected or sparsely linked, thereby highlighting areas of potential strength or vulnerability.

In practical terms, a positive Forman–Ricci curvature value indicates a node resides in a tightly knit cluster with robust interconnections, suggesting stability and resilience against external shocks. Conversely, a negative curvature value signifies that a node is in a more loosely connected or peripheral position, which may imply susceptibility to disruptions or a role as a bridge between disparate network clusters. By analyzing these curvature values across different nodes, we can discern patterns of connectivity that traditional centrality measures might overlook [31,32,33].

The combined effect of mutual information, TMFG filtering, and curvature-based metrics offers a layered analytical view—ranging from micro-level interactions (k=1) to larger-scale interdependencies (k=2 and k=3)—and thereby helps uncover the shifting architecture of each sector’s internal relationships. This multi-scale approach allows for the detection of both local and global structural changes within the network, facilitating a comprehensive analysis of how individual firms and entire sectors respond to economic stimuli and shocks.

To monitor these evolving structures over time, we apply a sliding window scheme, using windows of 15 trading days that advance by one day at a time, covering the period from early 2022 to the close of 2024. This dynamic design captures short-run fluctuations in market linkages while also allowing us to observe how persistent forces—such as high and volatile inflation, abrupt interest rate hikes, and episodic currency devaluations—reshape the internal organization of each sector. The chosen window size balances the need for sufficient data to compute reliable mutual information estimates with the necessity of maintaining temporal resolution to detect rapid changes.

Parallel to these empirical analyses, we map the curvature and its associated entropy to a timeline of notable macroeconomic events in Turkey: surging inflation, sizable currency depreciations, fluctuating interest rates, and apparent shifts in monetary policy orientation. By aligning our quantitative metrics with real-world events, we aim to contextualize the network dynamics within the broader economic environment. As each sector grapples with these forces, the geometric and entropy metrics help reveal whether firms cluster together or become more isolated, whether local firm-to-firm linkages weaken or broaden, and how systemic shocks ripple through broader clusters. This link between quantitative findings and real-world economic highlights situates the study’s results in a robust context, illuminating not just how the network evolves but also why certain shifts occur.

Furthermore, our approach allows for the identification of leading indicators of market stress and resilience. For instance, changes in curvature could signal emerging vulnerabilities within a sector before they manifest in price movements, providing valuable foresight for investors and policymakers alike. Entropy measures, reflecting the degree of disorder or uncertainty within the network, offer insights into the overall stability of sectoral interactions, with higher entropy indicating more complex and potentially less predictable relationships.

By illustrating sector-specific vulnerabilities and cross-sectoral channels of contagion or adaptation, this work expands existing market microstructure and systemic risk models, particularly within an emerging market context. Traditional correlation-based approaches, while still valuable, often mask crucial nonlinear behavior and may fail to depict how subtle connectivity shifts arise among different subsets of firms. In contrast, our geometric lens captures these subtleties, revealing where pockets of network fragility develop and where “hubs” in each sector’s connectivity might anchor resilience. Moreover, the extension of curvature analysis to different k-neighborhoods—paired with dynamic entropy calculations—offers a richer palette of indicators that can detect leading signals of stress or incipient realignments within a sector.

The implications of our findings extend beyond academic interest, holding practical relevance for a range of stakeholders. For policymakers, understanding the intricate network dynamics can inform more targeted and effective interventions aimed at stabilizing key sectors or mitigating systemic risks. For investors, the insights derived from our analysis can enhance portfolio diversification strategies and risk management practices by highlighting hidden dependencies and potential contagion pathways. Additionally, financial analysts and researchers can build upon our methodological framework to explore similar dynamics in other emerging markets, contributing to a more nuanced global understanding of financial stability and market behavior.

As policymakers and investors seek to navigate an environment marked by ongoing policy experimentation and fragile global conditions, these insights hold practical relevance. They suggest that the evolution of a sector’s network geometry is not merely an academic curiosity but a potent reflection of how firms co-adapt to a rapidly shifting macroeconomic backdrop. By providing a deeper understanding of the structural and dynamic properties of financial networks, our study contributes to the development of more resilient and adaptive financial systems capable of withstanding the multifaceted challenges of the contemporary economic landscape.

This paper is structured as follows: Section 2 details the foundational components of our study. It begins with a dataset, where we describe the sources, characteristics, and preprocessing steps of the financial data used for analysis. Following this, network analysis outlines the methodological framework, including the application of information theory, network science techniques, and discrete geometry tools employed to construct and analyze the financial networks of the selected BIST sectors. Section 3 presents the empirical findings and interprets their implications. This section is divided into two main parts, which explore the interactions and dependencies within individual sectors and examine the relationships and contagion channels between different sectors. Through these subsections, we elucidate how sector-specific and inter-sector dynamics respond to macroeconomic fluctuations and policy changes. Finally, Section 4 synthesizes the key insights derived from our analysis, highlighting the study’s contributions to understanding financial market dynamics in emerging economies. This section also discusses the practical implications for policymakers and investors, as well as potential avenues for future research.

## 2. Materials and Methods

### 2.1. Dataset

The dataset comprises several key indices that serve as vital indicators of Turkey’s economic performance on Borsa Istanbul. These include the BIST Industrial Index (XUSIN), the BIST Services Index (XUHIZ), the BIST Technology Index (XUTEK), the BIST Banking Index (XUBANK), and the BIST Electricity Index (XELEKT). Each index acts as a barometer of sector-specific activity and economic health, capturing how different segments of the market interact and evolve over time. Notably, the XUHIZ index integrates companies from the BIST Wholesale and Retail Trade (XTCRT) sector, which is why a separate XTCRT index is not utilized. From 2022 through 2024, these indices have been consistently influenced by macroeconomic factors—such as inflation, monetary policy, and global economic trends—underscoring their importance in understanding the dynamics of the Turkish stock market.

The industrial sector, represented by the BIST Industrial Index, plays a foundational role in Turkey’s economic framework. It encompasses manufacturing and production activities that are critical for exports and domestic economic growth [34,35,36]. This sector demonstrates resilience and dynamism in the face of challenges such as supply chain disruptions and currency volatility. The industrial sector consistently adapts to inflationary pressures and fluctuating global demand, making its network characteristics key to understanding the structural adaptability of Turkey’s economy. Similarly, the electricity sector—represented by the BIST Electricity Index plays a crucial role in powering the economy [37,38]. It comprises companies involved in the generation, transmission, and distribution of electrical energy, which are essential for supporting industrial production and meeting daily consumption needs. The services sector, captured by the BIST Services Index, reflects the broad spectrum of economic activities such as retail, tourism, and professional services. This sector thrives on domestic consumer activity and international tourism, both of which are sensitive to macroeconomic variables like inflation and exchange rates [39,40]. Despite challenges such as high inflation, the services sector maintains significant influence over the economy through its interconnected and adaptive business dynamics. The ongoing recovery in consumer and tourism activities underscores its importance in sustaining economic momentum. Technology companies, represented by the BIST Technology Index, are central to Turkey’s push toward innovation and digital transformation. This sector drives the adoption of new technologies and contributes to value creation across industries. It benefits from global trends favoring digitalization while facing headwinds such as rising costs and economic uncertainties [41,42,43]. The technology sector’s network behavior highlights its role as a driver of innovation and a bridge between traditional industries and modern economic models. The banking sector, represented by the BIST Banking Index, is integral to Turkey’s financial system and overall economic activity. It functions as a key intermediary in credit distribution and monetary policy implementation, supporting businesses and consumers alike. In an environment of high inflation and monetary policy adjustments, the banking sector remains pivotal in maintaining economic stability and liquidity [44,45,46,47]. Its network position reflects its systemic importance and its influence on the interconnected dynamics of other sectors.

The timescale of the study spans from 2022 to 2024, divided into yearly quartiles to capture detailed temporal dynamics within and across XUSIN, XUHIZ, XUTEK, XUBANK, and XELKT. This three-year period is characterized by significant economic events and transitions in the Turkish economy, making it ideal for analyzing sectoral interdependencies and market behaviors through network curvature. By segmenting the study into quarterly intervals, the analysis benefits from high temporal resolution, allowing for the observation of short-term fluctuations and longer-term trends. Each quarter reflects specific macroeconomic conditions, such as policy changes, inflation trends, currency movements, and sectoral performances. This granularity enables the study to capture the evolving relationships between sectors over time. The 2022 period includes the post-pandemic recovery phase and the beginning of pronounced inflationary pressures, offering insights into how sectors respond to global and domestic economic challenges. The quarterly breakdown for this year highlights how supply chain disruptions, monetary policy adjustments, and currency depreciation impact different sectors. The 2023 period represents a critical juncture, with continued inflationary pressures, policy responses, and signs of economic stabilization. Quarterly analysis here reveals the effectiveness of monetary tightening measures, sector-specific resilience, and changes in investor sentiment, providing a dynamic view of the Turkish stock market during an evolving macroeconomic environment. The 2024 period focuses on the stabilization efforts and outcomes of high-interest-rate policies, with particular attention to inflationary trends, industrial activity, and sectoral adjustments. Analyzing quarterly data during this time allows for detailed observation of how sectors adapt to sustained high interest rates, declining inflation, and potential shifts in economic sentiment. By examining data at this quarterly scale, the study ensures that sectoral responses to economic shocks, policy measures, and market trends are captured with high fidelity. This temporal framework enhances the ability to detect shifts in network structures, understand the resilience of specific sectors, and explore the evolution of intersectoral relationships over time. The economic details for each year’s quartiles are outlined in Table 1 while the descriptive statistics of the companies in each sector are presented in Table A1, Table A2, Table A3, Table A4 and Table A5.

In our study, data are collected from multiple reliable financial data providers, including Yahoo Finance, Investing, and Google Finance. We cross-check the datasets from these sources to ensure consistency and accuracy. Any discrepancies are carefully resolved through a rigorous validation process, resulting in a comprehensive dataset that contains no missing values. The preprocessing stage is streamlined by computing logarithmic returns from the raw price data, which inherently addresses concerns related to stationarity and outlier effects. By transforming price levels into log returns, the data not only becomes more stationary—a crucial requirement for many time-series analyses—but also mitigates the influence of extreme values.

### 2.2. Network Analysis

A sliding window framework is employed to examine how interactions among financial instruments evolve over time. Suppose we have a collection of *N* instruments (e.g., companies, assets, or financial indices) whose logarithmic returns are observed over a time series of length *T*. Denote by $r_{i}\left(t\right)$ the logarithmic return of the *i*-th instrument at the discrete time index *t*, where 1≤i≤N and 1≤t≤T. For illustration, let us assume that $r_{i}\left(t\right)$ is given by

(1) $$r_{i}\left(t\right)=\ln\left(P_{i}\left(t\right)\right)-\ln\left(P_{i}\left(t-1\right)\right),$$

where $P_{i}\left(t\right)$ is the (cleaned and validated) closing price of the *i*-th instrument on day *t*. In order to study how the structure of the financial market changes over time, we define a window size *W* (e.g., 15 trading days) and a step size *S* (e.g., 1 trading day). For the choice of the window size, please see Appendix B. Each window will thus encapsulate *W* consecutive observations for each of the *N* instruments, and we slide this window forward by *S* observations to obtain the next window.

Formally, let us define the *k*-th window $W_{k}$ as

(2) $$W_{k}=\left\{t|\;t_{0}+\left(k-1\right)S\leq t<t_{0}+\left(k-1\right)S+W\right\},$$

where $t_{0}$ is the index (e.g., $t_{0}=1$) marking the beginning of the time series, and *k* takes integer values in the range 1≤k≤K. The maximum number of windows, *K*, is given by

(3) $$K=\left\lfloor \frac{T-W+S}{S}\right\rfloor ,$$

ensuring that we do not exceed the length *T* of the entire time series. For each *k*, the set $W_{k}$ contains exactly *W* consecutive time points

(4) $$W_{k}=\left\{t_{0}+\left(k-1\right)S,t_{0}+\left(k-1\right)S+1,\ldots ,t_{0}+\left(k-1\right)S+W-1\right\}.$$

Using $W_{k}$, we construct a sub-matrix of returns, $R^{\left(k\right)}$, of dimension N×W. The (i,τ)-th entry of $R^{\left(k\right)}$ is the return $r_{i}\left(t\right)$ for instrument *i* at time *t*, with

(5) $$t=t_{0}+\left(k-1\right)S+\left(\tau -1\right),\;\mathrm{where}\;1\leq \tau \leq W\;\mathrm{and}\;1\leq i\leq N.$$

Hence,

(6) $$R^{\left(k\right)}=\left[\begin{array}{cccc} r_{1}\;\left(t_{0}+\left(k-1\right)S\right) & r_{1}\;\left(t_{0}+\left(k-1\right)S+1\right) & \cdots & r_{1}\;\left(t_{0}+\left(k-1\right)S+W-1\right)\\ r_{2}\;\left(t_{0}+\left(k-1\right)S\right) & r_{2}\;\left(t_{0}+\left(k-1\right)S+1\right) & \cdots & r_{2}\;\left(t_{0}+\left(k-1\right)S+W-1\right)\\ \vdots & \vdots & \ddots & \vdots \\ r_{N}\;\left(t_{0}+\left(k-1\right)S\right) & r_{N}\;\left(t_{0}+\left(k-1\right)S+1\right) & \cdots & r_{N}\;\left(t_{0}+\left(k-1\right)S+W-1\right) \end{array}\right].$$

This matrix $R^{\left(k\right)}$ represents the returns of all *N* instruments during the window $W_{k}$.

Mutual Information (MI) serves as a measure of dependence (particularly, nonlinear dependence) between any two time series [48,49]. Let $X_{i}$ and $X_{j}$ be the random variables corresponding to the returns of instruments *i* and *j* over $W_{k}$. We collect their returns into vectors

(7) $$r_{i}^{\left(k\right)}={\left[r_{i}\left(t_{0}+\left(k-1\right)S\right),\ldots ,r_{i}\left(t_{0}+\left(k-1\right)S+W-1\right)\right]}^{T},$$

(8) $$r_{j}^{\left(k\right)}={\left[r_{j}\left(t_{0}+\left(k-1\right)S\right),\ldots ,r_{j}\left(t_{0}+\left(k-1\right)S+W-1\right)\right]}^{T}.$$

Under the assumption that these entries approximate samples from random variables $X_{i}$ and $X_{j}$, Mutual Information $\mathrm{MI}(X_{i},X_{j})$ captures how much knowledge of $X_{j}$ reduces the uncertainty about $X_{i}$, and vice versa. In a general continuous setting, if p(x,y) is the joint probability density function (PDF) of $\left(X_{i},X_{j}\right)$, and p(x) and p(y) are the marginal PDFs of $X_{i}$ and $X_{j}$, respectively, then MI is given by

(9) $$\mathrm{MI}\left(X_{i},X_{j}\right)=\int_{X}\int_{Y}p\left(x,y\right)\ln\;\left(\frac{p(x,y)}{p(x)\;p(y)}\right)\;dx\;dy.$$

This integral is typically challenging to compute directly. In this study, a regression-based approach with *k*-neighborhood of 3 is adopted to approximate $\mathrm{MI}(X_{i},X_{j})$. Concretely, we can conceptualize the estimate as

(10) $$\hat{\mathrm{MI}}\left(X_{i},X_{j}\right)=\hat{I}\left(r_{i}^{\left(k\right)},\;r_{j}^{\left(k\right)}\right),$$

where $\hat{I}\left(\cdot \right)$ denotes an estimator function, which might involve local density approximations or regression-based variance decompositions. If $\hat{\mathrm{MI}}\left(X_{i},X_{j}\right)$ is large, it implies that $X_{i}$ and $X_{j}$ exhibit strong dependence (potentially capturing nonlinear co-movements in returns), whereas values near zero suggest near-independence of their dynamics in the chosen window.

Once all pairs (i,j) are processed, we construct an N×N mutual information matrix for the *k*-th window, denoted $M^{\left(k\right)}$. Formally, define

(11) $$M_{ij}^{\left(k\right)}=\left\{\begin{array}{cc} \hat{\mathrm{MI}}\left(X_{i},X_{j}\right), & i\neq j,\\ 0, & i=j, \end{array}\right.$$

where the diagonal entries are set to zero for convenience. This matrix captures all pairwise interdependencies among the *N* instruments during $W_{k}$. Repeating this process for each sliding window k=1,2,…,K yields a sequence of MI matrices

(12) $$\left\{M^{\left(1\right)},M^{\left(2\right)},\ldots ,M^{\left(K\right)}\right\},$$

thereby providing a temporal mapping of how the relationships among instruments evolve in response to market fluctuations, news events, or any other external forces influencing trading behaviors. Each $M^{\left(k\right)}$ can be interpreted as a fully connected weighted graph, where nodes represent instruments and edge weights reflect the strength of (nonlinear) dependence between their returns in the current window. This evolving network description sets the foundation for subsequent network filtration and curvature-based analyses that aim to capture the core structural properties of financial markets.

Continuing from the construction of the sliding-window-based mutual information (MI) matrices, each matrix $M^{\left(k\right)}$ for k∈{1,2,…,K} can be viewed as a fully connected, weighted graph $G^{\left(k\right)}=\left(V,E^{\left(k\right)}\right)$ with |V|=N and $|E^{\left(k\right)}|=\frac{N(N-1)}{2}$. Specifically, each node $v_{i}\in V$ corresponds to an instrument i∈{1,…,N}, while each edge $\left(v_{i},v_{j}\right)\in E^{\left(k\right)}$ carries a weight given by $M_{ij}^{\left(k\right)}=\hat{\mathrm{MI}}\left(X_{i},X_{j}\right)$. Formally, we denote

(13) $$w_{ij}^{\left(k\right)}=M_{ij}^{\left(k\right)},\;\mathrm{for}\;\mathrm{all}\;i\neq j,\;1\leq i,j\leq N,$$

indicating that the edge weight $w_{ij}^{\left(k\right)}$ represents the estimated mutual information between the returns of instruments *i* and *j* over the window $W_{k}$.

Since each matrix $M^{\left(k\right)}$ captures potentially high-dimensional dependencies (with $O(N^{2})$ edges), further network filtration steps are often necessary to reveal the most significant or structurally relevant edges. A widely used filtration approach in this context is the Triangulated Maximally Filtered Graph (TMFG), wherein edges with higher weights $w_{ij}^{\left(k\right)}$ are preferentially retained under constraints of planarity and triangulation. More precisely, one arranges the edges $\left\{(v_{i},v_{j})\right\}$ in descending order of $w_{ij}^{\left(k\right)}$ and subsequently attempts to insert them into an initially empty planar graph $H^{\left(k\right)}$. The insertion of each edge $\left(v_{i},v_{j}\right)$ is only accepted if it does not destroy the graph’s planarity and does not introduce chordless cycles of length greater than 3. Mathematically, this can be checked through a planarity test for Euler’s formula

(14) $$|V_{H^{\left(k\right)}}\left|-\right|E_{H^{\left(k\right)}}\left|+\right|F_{H^{\left(k\right)}}|=2,$$

where $|F_{H^{\left(k\right)}}|$ is the number of faces in the planar embedding of $H^{\left(k\right)}$, and through verifying that any newly formed cycle is at most triangle-length. As a consequence, the final TMFG preserves only the top-weighted edges while maintaining planarity and maximal triangulation, effectively summarizing the essential dependencies among instruments in each time window.

Once the TMFG filtration is complete, one obtains an adjacency matrix $A_{\mathrm{TMFG}}^{\left(k\right)}$ of size N×N, where

(15) $$A_{\mathrm{TMFG}}^{\left(k\right)}\left(i,j\right)\;=\;\left\{\begin{array}{cc} w_{ij}^{\left(k\right)} & \mathrm{if}\;\left(v_{i},v_{j}\right)\in E_{H^{\left(k\right)}}\\ 0 & \mathrm{otherwise}. \end{array}\right.$$

This yields a sparser, more interpretable graph $H^{\left(k\right)}$ representing each sector’s short-term network structure. By examining how $H^{\left(k\right)}$ changes with *k*, one can discern evolving patterns of market dependence, identify periods where certain edges (i.e., relationships) become more or less prominent, and gauge the overall connectivity or fragility of the sector.

In subsequent steps, curvature-based analyses (e.g., Forman–Ricci curvature) can be applied to each filtered network $H^{\left(k\right)}$ to probe deeper into the local geometric features of the graph. Such features include how “concentrated” or “spread out” certain clusters of nodes become, which in turn may reveal underlying systemic risks or highlight groupings of instruments moving in a highly correlated manner. Ultimately, combining the sliding window approach with TMFG filtration and curvature analytics furnishes a dynamic, geometry-aware perspective on financial networks, enabling researchers and analysts to better understand and monitor the structural shifts in these complex systems over time.

Recall that $H^{\left(k\right)}$ is a planar, triangulated graph with vertex set $V_{H^{\left(k\right)}}=\left\{v_{1},v_{2},\ldots ,v_{N}\right\}$ and edge set $E_{H^{\left(k\right)}}\subseteq E^{\left(k\right)}$. Let $w_{ij}^{\left(k\right)}$ denote the weight on edge $\left(v_{i},v_{j}\right)$ in $H^{\left(k\right)}$, which is inherited from the mutual information matrix $M^{\left(k\right)}$. In Forman’s discrete setting, each edge $e=\left(v_{i},v_{j}\right)\in E_{H^{\left(k\right)}}$ is equipped with an edge weight ω(e). Often, one lets $\omega \left(e\right)=w_{ij}^{\left(k\right)}$ or a monotonic function of $w_{ij}^{\left(k\right)}$. Let $\deg(v_{i})$ be the degree (valence) of node $v_{i}$ in $H^{\left(k\right)}$, and let F(e) denote the set of faces *f* (triangles in a planar, triangulated graph) that contain the edge *e*.

For weighted edges, the Forman–Ricci curvature $\kappa_{F}\left(e\right)$ of an edge *e* can be expressed as

(16) $$\kappa_{F}\left(e\right)=\omega \left(e\right)\;\left[\frac{\deg(i)}{\omega (e_{i})}+\frac{\deg(j)}{\omega (e_{j})}-2\right]-\sum_{f\;\in \;F(e)}\omega \left(f\right),$$

where ω(e) represents the weight of the edge *e*, and deg(i) and deg(j) are the weighted degrees of the nodes *i* and *j*, calculated as the sum of the weights of edges incident to these nodes. The terms $\omega (e_{i})$ and $\omega (e_{j})$ correspond to the weights of edges connected to *i* and *j*, respectively, while ω(f) denotes the weight of a face (triangular cycle), often defined in relation to the weights of its constituent edges. This formulation incorporates edge weights to modify the contributions of node degrees, ensuring that the curvature accurately reflects the influence of edge weights on the local structure of the graph. While different variants of Forman–Ricci curvature exist, depending on how node, edge, and face weights are integrated, the curvature $\kappa_{F}\left(e\right)$ consistently provides a localized measure of the graph’s geometry. A higher positive curvature indicates a more compact, denser structure around the edge, whereas a lower or negative curvature suggests a more dispersed or tree-like configuration.

Node-level curvature $\kappa_{F}\left(v_{i}\right)$ can be obtained by averaging the edge curvatures incident on $v_{i}$, i.e.,

(17) $$\kappa_{F}\left(v_{i}\right)=\frac{1}{\deg(v_{i})}\sum_{\left(v_{i},v_{j}\right)\in E_{H^{\left(k\right)}}}\kappa_{F}\left(v_{i},v_{j}\right).$$

Hence, each node $v_{i}$ is assigned a single scalar curvature value reflecting how the edges in its immediate neighborhood contribute to local geometric “tightness”.

To quantify the heterogeneity of node-level curvature across a local region of the network, one can define *k*-neighborhoods $N_{k}\left(v_{i}\right)$ for each node $v_{i}$. Formally,

(18) $$N_{k}\left(v_{i}\right)=\left\{v_{j}\in V_{H^{\left(k\right)}},d\left(v_{i},v_{j}\right)\leq k\right\},$$

where $d(v_{i},v_{j})$ denotes the shortest path distance (in edge count) between $v_{i}$ and $v_{j}$. Let $\kappa_{F}\left(v_{j}\right)$ be the curvature of node $v_{j}$. Then for the set $\left\{\kappa_{F}\left(v_{j}\right)\;|\;v_{j}\in N_{k}\left(v_{i}\right)\right\}$, we form a discrete probability distribution by shifting (to handle potential negative curvature) and normalizing the values: Let $\kappa_{\min}=\min_{v_{j}\in N_{k}\left(v_{i}\right)}\kappa_{F}\left(v_{j}\right)$. Define

(19) $${\tilde{\kappa }}_{F}\left(v_{j}\right)=\kappa_{F}\left(v_{j}\right)-\kappa_{\min}+\varepsilon ,$$

with a small ε>0 to avoid zero entries. The sum of shifted values is

(20) $$Z=\sum_{v_{j}\in N_{k}\left(v_{i}\right)}{\tilde{\kappa }}_{F}\left(v_{j}\right).$$

Hence, each node $v_{j}\in N_{k}\left(v_{i}\right)$ has probability

(21) $$p_{j}=\frac{{\tilde{\kappa }}_{F}\left(v_{j}\right)}{Z}.$$

The local curvature entropy $H_{k}\left(v_{i}\right)$ at node $v_{i}$ for the *k*-neighborhood is then given by the Shannon entropy of $\left\{p_{j}\right\}$

(22) $$H_{k}\left(v_{i}\right)=-\sum_{v_{j}\in N_{k}\left(v_{i}\right)}p_{j}\ln\left(p_{j}\right).$$

This metric $H_{k}\left(v_{i}\right)$ measures how diverse or concentrated the curvature values are in the immediate surroundings of $v_{i}$. If all $\kappa_{F}\left(v_{j}\right)$ are identical (implying a uniform local geometry), the entropy is low. Conversely, a wide spread of curvature values yields higher entropy, indicating a more structurally heterogeneous neighborhood.

To generate a global or sector-wide curvature-entropy descriptor for the entire TMFG $H^{\left(k\right)}$, one averages $H_{k}\left(v_{i}\right)$ over all nodes $v_{i}\in V_{H^{\left(k\right)}}$

(23) $${\bar{H}}_{k}=\frac{1}{|V_{H^{\left(k\right)}}|}\sum_{v_{i}\in V_{H^{\left(k\right)}}}H_{k}\left(v_{i}\right),\;k=1,2,3.$$

Thus, for each time window *k*, the triple $\left({\bar{H}}_{1},\;{\bar{H}}_{2},\;{\bar{H}}_{3}\right)$ encapsulates the average structural variability in the local geometry at distances 1, 2, and 3 around each node. By plotting or tabulating $\left({\bar{H}}_{1},\;{\bar{H}}_{2},\;{\bar{H}}_{3}\right)$ across all windows, one obtains a temporal map of the network’s geometric complexity, shedding light on how sector-level interdependencies strengthen or weaken and how local substructures evolve, possibly correlating with external market events or economic cycles.

## 3. Results and Discussion

### 3.1. Intersectoral Results

**Figure 1:**
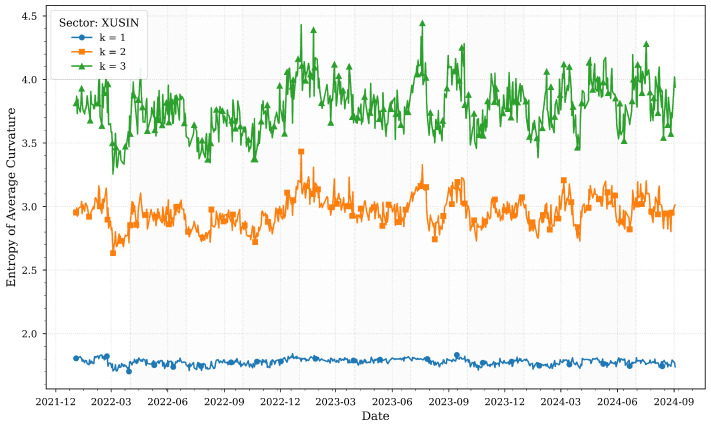
Forman–Ricci curvature entropies for various *k*-neighborhoods of the XUSIN sector across quartiles.

Figure 1 illustrates the computed entropies of average Forman–Ricci curvatures for various *k*-neighborhoods of the XUSIN sector across quartiles.

The entropy of average Forman–Ricci curvatures for the industrial sector (XUSIN) reflects its pivotal role in Turkey’s economy and its sensitivity to macroeconomic stressors. In early 2022, entropy values remain relatively stable, consistent with the strong post-pandemic recovery phase highlighted in Table 1, where Turkey’s economy grew by 5.5%. However, as inflation surged to a 20-year high of 72.3% in June 2022, entropy sharply declined, particularly in local neighborhoods (k=1). This drop suggests a reduction in structural complexity as firms faced synchronized pressures from rising input costs, currency depreciation, and supply chain disruptions.

By late 2023, entropy stabilized at higher levels, especially for broader neighborhoods (k=2,3), coinciding with monetary tightening measures and revised growth projections. This stabilization indicates that the sector adapted to high interest rates and declining inflation by diversifying linkages and mitigating risks. The resilience of the industrial sector during this period underscores its foundational role in Turkey’s export-driven economy, as noted in Table 1, where manufacturing and exports remained key drivers of growth despite challenges.

**Figure 2:**
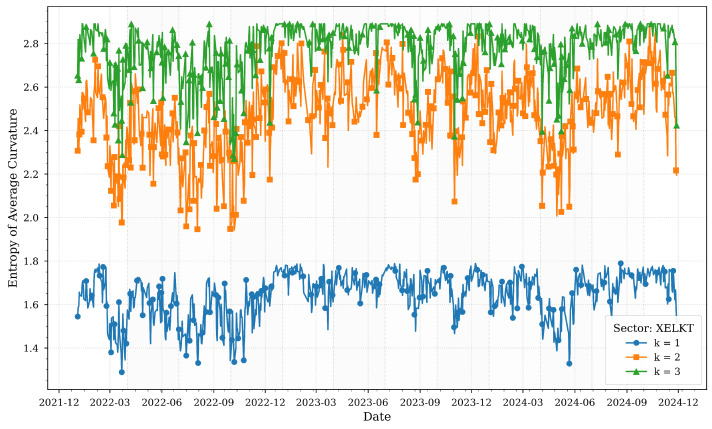
Forman–Ricci curvature entropies for various *k*-neighborhoods of the XELKT sector across quartiles.

Figure 2 illustrates the computed entropies of average Forman–Ricci curvatures for various *k*-neighborhoods of the XELKT sector across quartiles.

The electricity sector (XELKT) stands out for its remarkable stability across all k-neighborhoods, reflecting its critical role in powering the economy. Despite significant macroeconomic volatility—such as the 2022 currency crisis and the central bank’s aggressive interest rate hikes to 50% in 2024-entropy values for XELKT remain relatively flat. This consistency aligns with the sector’s essential function in supporting industrial production and meeting daily consumption needs.

Minor entropy dips in late 2023 correlate with reduced industrial capacity utilization (75.9% in Q3 2024), likely due to temporary demand-side adjustments. However, the overall low entropy variability reinforces the sector’s robust interconnections and systemic importance, acting as a buffer against transient shocks. The electricity sector’s stability highlights its resilience to external pressures, underscoring its role as a structural anchor in Turkey’s financial ecosystem.

**Figure 3:**
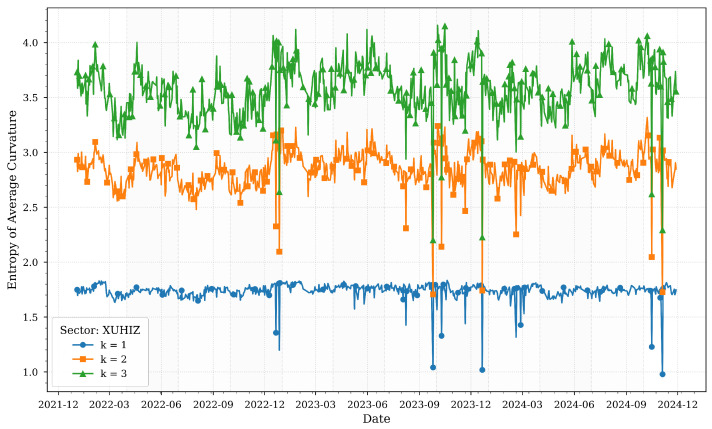
Forman–Ricci curvature entropies for various *k*-neighborhoods of the XUHIZ sector across quartiles.

Figure 3 illustrates the computed entropies of average Forman–Ricci curvatures for various *k*-neighborhoods of the XUHIZ sector across quartiles.

The services sector (XUHIZ) exhibits pronounced entropy volatility, particularly in local neighborhoods (k=1), reflecting its sensitivity to consumer sentiment and macroeconomic fluctuations. Peaks in early 2023 correspond to inflationary pressures and shifts in consumer demand, where rapid adjustments in retail and tourism networks increased structural unpredictability. This aligns with Table 1, which notes that private consumption and exports drove economic expansion during this period.

The entropy decline in mid-2023 coincides with monetary policy normalization, suggesting tighter clustering of firms as credit conditions stabilized. By 2024, broader neighborhoods (k=3) show rising entropy, reflecting the sector’s role as an intermediary transmitting shocks between banking and industrial/technology sectors. This mirrors the study’s cross-correlation analysis, which identifies services as a conduit for macroeconomic disturbances. The sector’s adaptive business dynamics during economic stabilization further underscore its importance in sustaining economic momentum.

**Figure 4:**
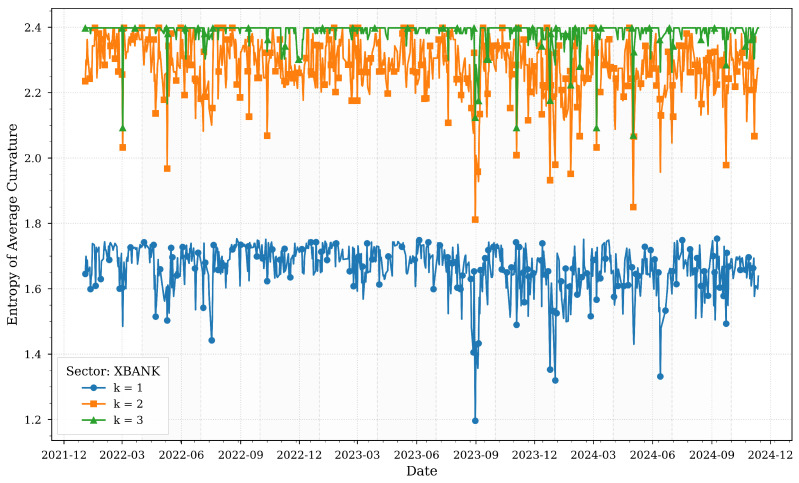
Forman–Ricci curvature entropies for various *k*-neighborhoods of the XUTEK sector across quartiles.

Figure 4 illustrates the computed entropies of average Forman–Ricci curvatures for various *k*-neighborhoods of the XUTEK sector across quartiles.

The technology sector (XUTEK) reveals episodic spikes in entropy, notably in late 2022 and mid-2024. The 2022 surge aligns with global tech-driven market corrections and domestic financing constraints, amplifying structural heterogeneity as firms grappled with innovation cycles and funding uncertainties. This period corresponds to the inflation surge and currency depreciation highlighted in Table 1, which likely exacerbated these challenges.

A sharp entropy drop in early 2024 coincides with the central bank’s aggressive rate hikes (50%), which may have streamlined network dependencies as firms prioritized liquidity. However, rising entropy in broader neighborhoods (k=3) by late 2024 suggests renewed complexity driven by digital transformation efforts and cross-sector integration with industrial and services firms. These trends reflect the sector’s dual exposure to global innovation cycles and domestic financing conditions, as noted in the study’s cross-correlation analysis.

**Figure 5:**
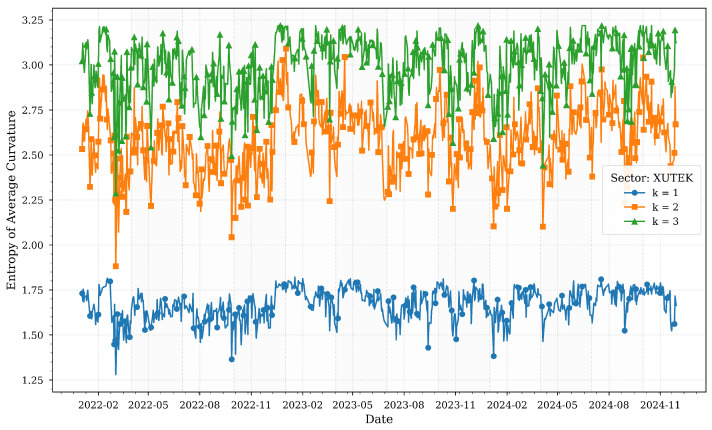
Forman–Ricci curvature entropies for various *k*-neighborhoods of the XBANK sector across quartiles.

Figure 5 illustrates the computed entropies of average Forman–Ricci curvatures for various *k*-neighborhoods of the XBANK sector across quartiles.

The banking sector (XBANK) demonstrates entropy dynamics that reflect its systemic role in Turkey’s financial ecosystem. Local neighborhoods (k=1) exhibit heightened entropy during the 2022 currency crisis and 2023 monetary policy shifts, indicating fragmented responses to liquidity shocks. This aligns with Table 1, which highlights the banking sector’s centrality in credit distribution and monetary policy implementation.

However, broader neighborhoods (k=3) show stabilizing entropy by 2024, coinciding with sustained high interest rates (50%) and declining inflation (47.09% in November 2024). This stabilization suggests that while individual banks faced localized stress, systemic interdependencies strengthened, anchoring resilience. The sector’s entropy patterns underscore its dual role as both a transmitter and absorber of macroeconomic shocks, consistent with its influence on interconnected dynamics across other sectors.

The interplay between entropy trends and macroeconomic events in Table 1 highlights sector-specific vulnerabilities and adaptive mechanisms. For instance, the XUSIN and XUHIZ sectors, sensitive to inflation and consumer demand, exhibited entropy fluctuations mirroring policy shifts and external shocks. The stability of XELKT and the systemic resilience of XBANK underscore their foundational roles in Turkey’s economy. The entropy patterns of XUTEK reflect its dual exposure to global innovation cycles and domestic financing conditions. Together, these findings validate the utility of curvature-based entropy in capturing nuanced sectoral responses to Turkey’s turbulent economic landscape.

### 3.2. Cross-Sectoral Results

In the ever-evolving financial markets, gaining insights into the complex dynamics and interdependencies among different sectors is essential for informed decision-making and strategic planning. We assess these connections by employing a suite of analytical techniques, including cross-correlation with lags, Granger causality tests [50], and transfer entropy [51] to capture both linear and nonlinear dependencies. Cross-correlation analysis with lags enables us to identify lead-lag relationships between sectors, revealing how fluctuations in one area may precede or follow changes in another and thereby uncovering potential causal sequences and synchronization patterns. Granger causality tests further evaluate whether historical values of one sector’s curvature measure possess predictive power over another, establishing directional influences and shedding light on underlying causative relationships. Meanwhile, transfer entropy quantifies the directional flow of information by measuring the reduction in uncertainty when the past behavior of one sector is used to predict the future dynamics of another, effectively capturing nonlinear interactions that might otherwise be overlooked.

**Figure 6:**
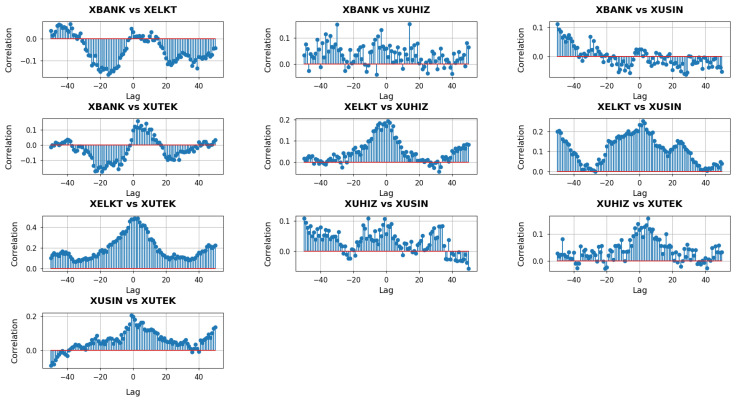
Cross-correlation analysis with lag of Forman–Ricci curvature entropies for k=1 between sectors.

Figure 6 depicts the cross-correlation analysis with lags for k=1. When correlations at negative lags are pronounced, it suggests that one sector’s curvature adjustments may systematically precede the corresponding shifts in another sector. Conversely, if significant correlations appear at positive lags, it is the latter sector whose network changes seem to lead. Around zero lag, visible peaks or troughs indicate simultaneous movements, implying that network geometries of both sectors react in unison to market conditions or policy developments.

In the XBANK vs. XUHIZ panel, the cross-correlation profile displays a broad mix of positive and negative values across the lag spectrum. This variability suggests that the banking and services sectors occasionally respond to macroeconomic events with slight timing offsets yet also align around zero lag when shared factors—such as consumer demand or liquidity conditions—drive both sectors simultaneously. In contrast, the XBANK vs. XUSIN panel features stronger positive correlations on the negative lag side that fade or turn negative for positive lags, indicating that banking-related adjustments often precede or coincide with subsequent shifts in industrial firms’ network structures. This pattern likely reflects how changes in financing availability and interest rates can directly influence capital-intensive industrial activities.

The XBANK vs. XUTEK cross-correlations stand out due to pronounced negative values at some negative lags, flipping to strong positive correlations near zero. Such behavior implies that the local network geometry of XUTEK can initially diverge from banking conditions—perhaps when credit tightening disproportionately affects smaller or more research-focused tech companies—before both sectors realign in response to broader market forces. Meanwhile, the XUHIZ vs. XUSIN panel shows moderate positive clusters at negative lags, suggesting that services may act as an early indicator for industrial shifts, particularly if evolving consumer preferences and demand feed into manufacturing decisions. The XUHIZ vs. XUTEK relationship exhibits large positive correlations concentrated around zero and slightly positive lags, pointing to periods when consumer-driven or digital trends cause both sectors to move in sync, although technology may occasionally trail services if policy or financial catalysts originate on the consumer side. Finally, the XUSIN vs. XUTEK panel reveals notably high correlations near zero lag, highlighting how closely manufacturing activities can track technological developments, leading both networks to adapt nearly in tandem whenever tech-driven solutions become integral to production processes.

The *p*-values obtained from the Granger causality test with k=1 across various lags are summarized in Table 2.

The table presents the *p*-values from the Granger causality tests for k=1, examining whether past changes in the curvature measure of one sector can predict subsequent changes in another. When we consider the relationship between XBANK and XELKT, the consistently high *p*-values across all lags suggest that variations in XBANK’s curvature do not significantly forecast shifts in XELKT. In contrast, the tests from XBANK to XUHIZ reveal a dramatic shift: while the *p*-value at lag 1 remains high, the values from lag 2 onward are extremely low, indicating that adjustments in the banking sector’s curvature reliably precede those in the services sector, possibly reflecting the influence of financing conditions on consumer-related dynamics.

Similarly, the causality tests from XBANK to both XUSIN and XUTEK sectors yield relatively high *p*-values, indicating that the banking sector does not exhibit significant leading effects on these sectors at the local level. However, when we shift our focus to the XELKT sector, its curvature appears to have a predictive role. The tests show that XELKT significantly Granger-causes changes in XUSIN with all *p*-values falling below conventional significance levels, and it even more strongly predicts changes in XUTEK, with *p*-values reaching zero or near zero across multiple lags. Furthermore, the analysis reveals that XUHIZ also exerts a strong causal influence on both the industrial and technology sectors, as demonstrated by consistently low *p*-values across most lags. Finally, although the XUSIN sector’s influence on the XUTEK sector is significant only at the shorter lags (lags 1 and 2), the overall picture is one of clear directional relationships, where certain sectors, particularly electricity and services, drive subsequent adjustments in others, highlighting the dynamic interplay within the financial network at the local scale.

The transfer entropy (TE) values in Table 3 for k=1 provide insight into the directional flow of information between pairs of sectors by quantifying how much knowing the past values of one sector reduces uncertainty about the future values of another. In this table, each row represents a unique pair of sectors, and two values are provided: one measuring the transfer entropy from the second sector (B) to the first (A) and the other from the first sector (A) to the second (B). These values, although generally small in magnitude, are significant in that they capture subtle nonlinear dependencies that traditional linear methods might overlook.

For example, in the comparison between XBANK and XELKT, the transfer entropy from XELKT to XBANK is 0.0271, while the transfer entropy from XBANK to XELKT is 0.0363. This indicates that there is a slightly stronger directional influence from XBANK to XELKT than vice versa, suggesting that changes in the banking sector’s network geometry may have a more pronounced effect on the electricity sector than the other way around. Similarly, when examining the relationship between XBANK and XUHIZ, the TE values of 0.0068 from XUHIZ to XBANK and 0.0109 from XBANK to XUHIZ point to a modest yet discernible information flow primarily from the banking sector to the services sector.

The asymmetries become more pronounced in other pairs as well. In the XBANK versus XUSIN pair, the transfer entropy is 0.0343 from XUSIN to XBANK compared to 0.0240 in the opposite direction, which implies that the industrial sector may exert a stronger influence on banking dynamics than the reverse. The TE values between XELKT and the other sectors also reveal notable directional differences. For instance, the transfer entropy from XELKT to XUHIZ is 0.0187 while the reverse is 0.0171, indicating a nearly balanced, albeit slightly higher, flow of information from electricity to services. The relationship between XELKT and XUTEK is even more intriguing; both directions show very low paces of transfer entropy with values of 0.0198 and 0.0149, respectively, suggesting that the interaction between the electricity and technology sectors is relatively weak or perhaps more reciprocal.

When the focus shifts to the services and industrial sectors (XUHIZ and XUSIN), the transfer entropy values of 0.0301 from XUSIN to XUHIZ and 0.0287 from XUHIZ to XUSIN demonstrate a strong bidirectional influence, indicating that these sectors are closely intertwined. The dynamics between XUHIZ and XUTEK, with values of 0.0230 and 0.0247, reinforce this notion of tight interdependence, where changes in one sector are almost immediately reflected in the other. Finally, the industrial and technology sectors (XUSIN and XUTEK) exhibit TE values of 0.0175 from XUTEK to XUSIN and 0.0189 in the opposite direction, suggesting a relatively balanced and moderate exchange of information.

**Figure 7:**
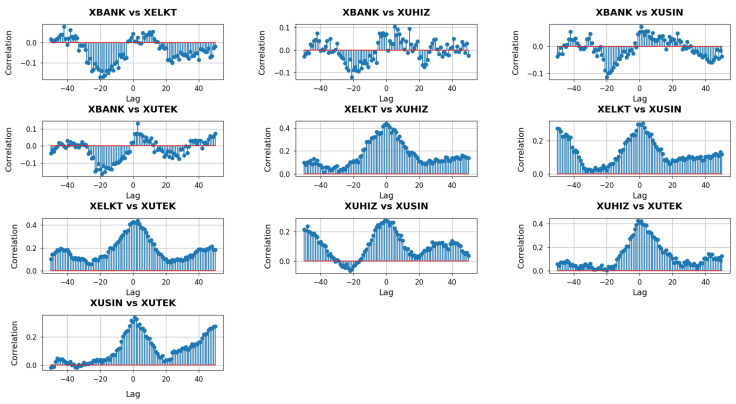
Cross-correlation analysis with a lag of Forman–Ricci curvature entropies for k=2 between sectors.

With k=2 capturing mid-range relationships in each sector’s network, these cross-correlation plots in Figure 7 provide a window into how broader—but not yet fully system-wide—structural shifts in one sector may precede, coincide with, or follow those of another.

At the mid-range neighborhood scale (k=), the cross-correlation plots reveal broader interconnections among the sectors than those observed at the local (k=1) level. In the panels involving XBANK, the correlation patterns show a mixture of positive and negative swings across negative and positive lags. This behavior indicates that while there are intervals where changes in one sector systematically precede those in the other, the timing is not strictly consistent across all windows. For instance, XBANK vs. XUHIZ exhibits some pronounced correlations around negative lags, suggesting that short-run adjustments in banking may sometimes lead the services sector. Conversely, near positive lags, the influence can invert or diminish, implying a dynamic feedback loop rather than a one-directional lead-lag relationship. In contrast, XBANK vs. XUSIN and XBANK vs. XUTEK present moderate correlation levels, with fewer distinct peaks, suggesting that banking’s mid-range interplay with industrial and tech firms, though present, is less sharply defined than its influence on services.

Focusing on the pairs that do not include XBANK highlights additional nuances. The XELKT vs. XUHIZ plot, for example, shows clusters of positive correlations centered around slightly negative lags, implying that when the electricity sector undergoes mid-range structural shifts, the services sector may soon follow. A similar pattern arises in XELKT vs. XUSIN, though with some intervals indicating that industrial changes can also anticipate electricity adjustments, reflecting the two-way influence of manufacturing demand and energy supply. Meanwhile, XELKT vs. XUTEK reveals modest but steady correlations around zero, indicating that both sectors can move in unison in response to overarching market signals, albeit without extreme spikes. The XUHIZ vs. XUSIN panel stands out for its strong positive correlations at negative lags, hinting that consumer-oriented shifts in services often herald broader changes in industrial output, whereas XUHIZ vs. XUTEK presents a noticeable peak near zero lag, suggesting that these two sectors frequently align when macroeconomic or policy factors prompt synchronized responses. Finally, XUSIN vs. XUTEK displays narrower fluctuations overall, signifying that while industrial and technology sectors do exhibit mid-range concurrency, their cross-correlation is less volatile, pointing to a relatively stable, though not overly dramatic, mutual influence.

The *p*-values obtained from the Granger causality test with k=2 across various lags are summarized in Table 4.

The Granger causality results at k=2 reveal that certain mid-range relationships among sectors become more pronounced than they appear at shorter horizons. When we look at XBANK, its predictive influence on other sectors is mixed. The *p*-values for XBANK to XELKT remain high at all tested lags, suggesting no significant causal effect. By contrast, XBANK to XUHIZ becomes significant from lag 2 onward, implying that once a short delay is accounted for, banking’s mid-range adjustments can reliably forecast changes in the services sector’s curvature. XBANK’s relationship with the XUSIN sector remains statistically insignificant across lags, indicating no consistent predictive power, while the *p*-values for XBANK to XUTEK show significance at lag 2 and lag 3 before becoming marginal at higher lags, suggesting a brief window where banking leads technology but does not sustain that influence over a longer period.

Examining XELKT highlights a notable predictive role for its mid-range curvature changes. XELKT exerts a significant influence on XUHIZ as early as lag 1, and this influence persists through multiple lags, suggesting that shifts in electricity’s network structure have meaningful downstream effects on service-oriented firms. The relationship with XUSIN is also significant at lag 1, though the *p*-values gradually rise as the lag lengthens, indicating a short-term influence that diminishes somewhat over time. For XUTEK, the significance remains just below conventional thresholds in the earlier lags and becomes borderline at later ones, hinting at a moderate yet not overwhelmingly strong predictive link between electricity and technology at mid-range intervals.

Turning to XUHIZ, the results show consistently low *p*-values when predicting XUSIN over several lags, underscoring how consumer-facing or service-based activities can lead to manufacturing trends, possibly through shifts in consumer demand or retail-driven supply chains. XUHIZ also demonstrates a strong, though slightly shorter-lived, influence on XUTEK, with *p*-values staying below 0.05 across lags 1 to 5, suggesting that services-based innovations or demand patterns can ripple through to tech firms. Finally, XUSIN displays a similarly significant relationship when predicting XUTEK, indicating that manufacturing-related developments may precede changes in the local network structure of XUTEK, possibly through the adoption of new production techniques or hardware demands.

TE values obtained from transfer entropy with k=2 are presented in Table 5.

Notably, the XELKT and XUHIZ pair exhibits the largest transfer entropy values in both directions, with 0.055475 from XELKT to XUHIZ and 0.049321 in the reverse direction. This near-symmetry suggests that shifts in the electricity sector’s mid-range structure can have a strong downstream impact on services, while service-oriented changes can also propagate back into the electricity network, possibly through demand-driven adjustments in power consumption and pricing.

XUHIZ and XUSIN pair registers transfer entropy values of 0.050213 and 0.048173, again underscoring the reciprocal nature of their mid-range dynamics. Such a pattern may emerge when consumer-driven shifts in the service sector influence manufacturing demand, which then circles back to shape service offerings or pricing. In XUTEK interactions, the magnitudes are slightly lower but remain relatively close between directions. For instance, XELKT to XUTEK is 0.033785 while XUTEK to XELKT is 0.036209, pointing to a moderate but still mutual interplay—likely reflecting how technological developments in software or hardware affect energy usage and grid modernization and how changes in energy infrastructure can open or constrain technological innovation.

Finally, while XBANK shows notable transfer entropy values with all sectors, these values tend to be more modest than the highest-scoring pairs. In some instances, the flow from banking to other sectors is marginally stronger, while in others, the opposite is true, indicating that financial conditions can both drive and be driven by developments in the real economy. Overall, the table suggests that mid-range information flows are especially prominent between electricity and services and between services and industrial, revealing how these pairs may serve as key conduits for sectoral adjustments in the broader market network.

**Figure 8:**
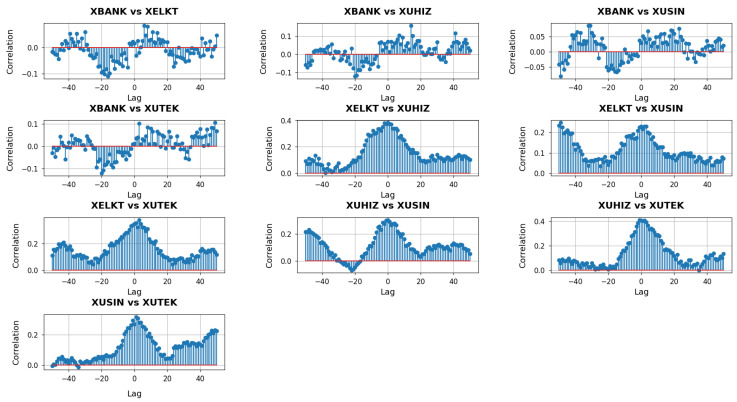
Cross-correlation analysis with lag of Forman–Ricci curvature entropies for k=3 between sectors.

With k=3 capturing the largest-scale connectivity in each sector’s network, these cross-correlation plots in Figure 8 show how system-wide shifts in one sector can lead, lag, or coincide with those in another.

At the widest neighborhood scale (k=3), the cross-correlation plots capture how the largest sets of interlinked nodes in each sector’s network respond to market forces and each other. In the panels involving XBANK, notably its comparisons with all other sectors, the correlation values tend to show broader fluctuations across both negative and positive lags. This behavior suggests that when the XBANK’s far-reaching connections shift, the other sectors sometimes follow, yet at other times, they appear to lead, indicating a two-way dynamic. For instance, in XBANK vs. XUHIZ, there may be a distinct band of positive correlation at negative lags, implying that structural changes in banking can foreshadow subsequent movements in services. Conversely, closer to zero or positive lags, the two sectors often align or invert, reflecting how consumer-facing services can, in turn, affect XBANK’s broader network structure.

The pairs that do not involve XBANK underscore additional facets of intersectoral interplay at this larger scale. XELKT and XUHIZ may show consistent positive correlations at a range of lags, highlighting how shifts in power generation, distribution, or pricing can propagate into consumer-driven segments of the market. When comparing XELKT and XUSIN, the correlation profile typically displays moderate peaks at negative or near-zero lags, suggesting that industrial production needs or capacity utilization may sometimes precede changes in the electricity sector, though the influence can reverse if energy constraints or regulatory changes impact manufacturing operations. In XELKT vs. XUTEK, the fluctuations can be relatively balanced, indicating that the broader energy-tech relationship often moves in tandem when large-scale factors—like infrastructure investments or digitalization strategies—take hold across multiple firms.

Turning to the XUHIZ and XUSIN pair, strong correlations at negative lags frequently imply that changes in consumer demand or retail dynamics can predate shifts in manufacturing, while near-zero lag peaks reflect moments when both sectors react jointly to overarching macroeconomic or policy shifts. XUHIZ and XUTEK similarly exhibit synchronized bursts of correlation, especially around zero lag, pointing to instances when widespread digital adoption or consumer-driven tech services spur nearly simultaneous adjustments in both networks. Finally, XUSIN and XUTEK may reveal a more modest but consistent correlation pattern, suggesting that large-scale technological advances do, at times, drive or follow industrial expansions.

The *p*-values obtained from the Granger causality test with k=3 across various lags are summarized in Table 6.

Examining XBANK, its relationship with XELKT and XUSIN remains insignificant across all tested lags, suggesting that even when considering broader network interactions, XBANK does not systematically lead these two sectors. In contrast, the *p*-values for XBANK to XUHIZ drop sharply from lag 2 onward, reinforcing the idea that once a short delay is accounted for, the banking sector’s large-scale adjustments can reliably forecast changes in services. Meanwhile, the borderline results for XBANK to XUTEK hint at some potential influence around lag 2 and lag 4, although the *p*-values remain above the conventional significance threshold, indicating no definitive lead-lag relationship at this scale.

Turning to XELKT, its predictive role appears strong when considering XUHIZ, with *p*-values near zero at the first two lags and staying below 0.05 at lags 3, 4, and 5 as well. This pattern suggests that large-scale shifts in electricity—possibly linked to regulation, infrastructure, or energy pricing—exert a measurable impact on services over multiple days. The relationships with XUSIN and XUTEK also show significance at earlier lags, though the *p*-values gradually approach or surpass 0.05 at higher lags, implying that while XELKT holds predictive power in the short to mid range, this influence tapers off over longer horizons.

XUHIZ continues to demonstrate considerable predictive strength with respect to both XUSIN and XUTEK. The *p*-values remain consistently below 0.05 up to lag 5, signifying that consumer-driven or service-oriented changes can systematically lead to broader industrial and tech responses. This pattern likely reflects the wide-reaching effect of shifts in consumer demand, retail patterns, or service innovations that eventually filter into manufacturing and technology adoption. Finally, XUSIN also exerts a notable influence on XUTEK, with *p*-values under 0.05 through all five lags.

TE values obtained from transfer entropy with k=3 are presented in Table 7.

Comparing the pairs involving XBANK with other sectors shows that XBANK’s transfer entropy levels tend to be lower in both directions. This suggests that while the banking sector does exchange information with others, it is not as deeply interwoven at the widest network level as some of the real-economy sectors are with each other.

In contrast, pairs like XELKT and XUHIZ stand out with high bidirectional transfer entropy values, especially from XELKT to XUHIZ (0.067199). This finding implies that large-scale changes in the electricity sector—potentially involving shifts in generation capacity, grid infrastructure, or pricing structures—can substantially reduce uncertainty about how services will evolve. XUHIZ, in turn, also exhibits a considerable influence on electricity, though to a slightly lesser degree. Similar patterns emerge among XELKT, XUSIN, and XUTEK, where moderate to high values in both directions underscore a robust, mutual exchange of information at the broader neighborhood scale. Such reciprocity likely reflects how shifts in industrial or technological processes can affect energy demand and supply while developments in electricity infrastructure or innovation feed back into manufacturing and digital ecosystems.

The relationships among XUHIZ, XUSIN, and XUTEK also display relatively balanced and moderately high transfer entropy values in both directions, pointing to a tight interplay at this wider scale. For instance, the flows between XUHIZ and XUSIN (0.043762 vs. 0.038213) indicate that consumer-driven demand or retail expansion can meaningfully inform industrial output and vice versa. Meanwhile, XUTEK interacts strongly with both XUSIN and XUHIZ, suggesting that the widespread adoption of new digital or production solutions has far-reaching implications that ripple across these sectors. Overall, the table reveals that at the largest neighborhood level, electricity, services, industrial, and technology are all closely intertwined, while banking exhibits more modest information flows—a pattern that highlights the systemic importance of energy, consumer demand, manufacturing, and technology innovation in shaping market-wide dynamics.

## 4. Conclusions

This study introduces an innovative network-geometric framework to analyze the dynamic interdependencies among key sectors of Borsa Istanbul, including industrial, services, technology, banking, and electricity. By transforming daily stock price data into logarithmic returns and employing a sliding window approach, we constructed weighted networks that highlight the most significant dependencies using Triangulated Maximally Filtered Graphs (TMFG). The application of Forman–Ricci curvature at the node level, combined with entropy measures across different neighborhood scales, provides a multidimensional perspective on both local and global connectivity patterns within these sectors. This methodology surpasses traditional correlation-based approaches, offering deeper insights into how market relationships evolve under varying macroeconomic conditions.

The intersectoral analysis reveals distinct structural characteristics that align closely with the unique economic roles and vulnerabilities of each sector. For instance, the industrial sector demonstrates heightened sensitivity to macroeconomic stressors such as currency depreciation and rising input costs, as demonstrated by pronounced dips in broader network measures during turbulent periods. In contrast, the services sector exhibits sharper fluctuations driven by shifts in consumer demand and spending behavior, underscoring its responsiveness to domestic economic conditions. The technology sector maintains a relatively cohesive network structure, though occasional sharp adjustments highlight its susceptibility to financing constraints and global innovation cycles. The banking sector, despite localized stress at the k=1 level, retains robust systemic interdependencies at broader scales, reflecting its pivotal role in maintaining liquidity and credit distribution. Meanwhile, the electricity sector strikes a balance between short-term local variations and a stable overall structure, indicative of its critical importance in powering the economy and its resilience to external shocks.

Cross-sectoral analyses further illuminate the intricate channels through which shocks propagate across markets. Techniques such as cross-correlation, Granger causality, and transfer entropy reveal that certain sectors—particularly services and electricity—act as conduits for transmitting macroeconomic disturbances to others. For example, the services sector emerges as a critical intermediary, channeling shocks between the banking and both the industrial and technology sectors. Similarly, the electricity sector’s stability often serves as a buffer against broader disruptions, while its vulnerabilities can ripple through interconnected industries. These findings underscore the interconnected nature of Turkey’s financial ecosystem, where no sector operates in isolation, and external shocks are amplified or mitigated through complex interdependencies.

While this study demonstrates the theoretical advantages of Forman–Ricci curvature and entropy measures in capturing subtle geometric properties of network structures, it is not without limitations. The exclusive reliance on daily closure prices may overlook finer-scale interactions that high-frequency data could reveal. Additionally, the empirical validation of these geometric metrics against traditional measures remains an area for future exploration. Incorporating alternative dependency measures or extending the analysis to include intraday volatility and volume-weighted returns could further substantiate the robustness of the proposed framework.

Looking ahead, translating these geometric insights into practical decision-making tools presents exciting opportunities. For instance, threshold-based strategies derived from historical analysis of Forman–Ricci curvature and entropy measures could serve as early warning systems for portfolio managers. A sustained decline in average curvature or a spike in entropy beyond predefined thresholds could trigger specific risk mitigation actions, such as reallocating investments or increasing hedging positions. Such strategies not only enhance risk management but also provide actionable guidelines for portfolio optimization based on the dynamic behavior of network metrics.

In summary, this research lays a robust foundation for understanding the complex interdependencies among financial sectors through the integration of network science, information theory, and discrete geometry. By tracking structural shifts at multiple neighborhood scales and linking these to macroeconomic developments, the study offers a nuanced perspective on how shocks propagate across markets. While further refinement and exploration with additional data are necessary, the present work contributes significantly to both academic inquiry and the development of actionable investment strategies informed by geometric measures. Future research should focus on validating these findings across diverse markets and exploring their applicability to other emerging economies, thereby advancing our understanding of financial stability and systemic risk in a global context.

## Figures and Tables

**Table 1:** Highlights of Turkey’s economy from 2022 to 2024, presented by quarterly periods, showcasing key trends such as inflation, monetary policy changes, and sectoral growth.

Time Period	Economic Highlights
2022: January–March	Turkey’s economy experienced a strong recovery, with a 5.5% growth rate for the year.
2022: April–June	Inflation rose to a 20-year high, reaching 72.3% by June, exacerbated by global supply constraints and buoyant domestic demand.
2022: July–September	The Turkish lira depreciated significantly, leading to increased import costs and further inflationary pressures.
2022: October–December	Despite high inflation, the economy continued to grow, with a focus on exports and manufacturing.
2023: January–March	The economy expanded by 4.5%, driven by private consumption and exports.
2023: April–June	Inflation remained high, posing challenges to economic stability.
2023: July–September	The central bank implemented monetary tightening measures to combat inflation, signaling a shift towards orthodox monetary policies.
2023: October–December	The economy showed signs of stabilization, with growth projections revised upwards.
2024: January–March	The central bank increased the interest rate to 50% under the new governor, aiming to control inflation.
2024: April–June	Inflation rates began to decline, with consumer prices rising by 75.45% in May, indicating a potential peak.
2024: July–September	The capacity utilization rate in the manufacturing industry decreased to 75.9%, indicating a slowdown in industrial activity.
2024: October–December	Inflation decreased to 47.09% in November, with the central bank maintaining interest rates at 50% for eight consecutive months, indicating efforts to stabilize the economy.

**Table 2:** *p*-values of Granger causality test for k=1.

Sectors		Lag 1	Lag 2	Lag 3	Lag 4	Lag 5
XBANK	XELKT	0.9189	0.4871	0.7947	0.6546	0.5924
XBANK	XUHIZ	0.9745	0.0001	0.0006	0.0005	0.0005
XBANK	XUSIN	0.2737	0.8684	0.9136	0.9747	0.9562
XBANK	XUTEK	0.1312	0.555	0.6277	0.77	0.9113
XELKT	XUHIZ	0.0079	0.0306	0.1286	0.1271	0.0761
XELKT	XUSIN	0.0	0.0026	0.008	0.0157	0.0219
XELKT	XUTEK	0.0	0.0	0.0	0.0001	0.0001
XUHIZ	XUSIN	0.0	0.0	0.0	0.0004	0.0006
XUHIZ	XUTEK	0.0016	0.0127	0.01	0.0268	0.011
XUSIN	XUTEK	0.0001	0.0102	0.1051	0.1834	0.2856

**Table 3:** Transfer Entropy values for k=1.

Sectors		TE from B to A	TE from A to B
XBANK	XELKT	0.027056	0.036255
XBANK	XUHIZ	0.006798	0.010898
XBANK	XUSIN	0.034267	0.023961
XBANK	XUTEK	0.025942	0.033149
XELKT	XUHIZ	0.018721	0.017051
XELKT	XUSIN	0.013567	0.012321
XELKT	XUTEK	0.019762	0.014896
XUHIZ	XUSIN	0.030123	0.028654
XUHIZ	XUTEK	0.022987	0.024671
XUSIN	XUTEK	0.017456	0.018923

**Table 4:** *p*-values of Granger causality test for k=2.

Sectors		Lag 1	Lag 2	Lag 3	Lag 4	Lag 5
XBANK	XELKT	0.9956	0.9309	0.9806	0.8178	0.8811
XBANK	XUHIZ	0.4551	0.0055	0.0099	0.0096	0.0180
XBANK	XUSIN	0.8543	0.3068	0.3022	0.4547	0.5763
XBANK	XUTEK	0.2711	0.0198	0.0428	0.0862	0.1256
XELKT	XUHIZ	0.0000	0.0002	0.0091	0.0045	0.0077
XELKT	XUSIN	0.0013	0.0124	0.0487	0.0775	0.0942
XELKT	XUTEK	0.0109	0.0241	0.0382	0.0523	0.0678
XUHIZ	XUSIN	0.0004	0.0021	0.0056	0.0093	0.0112
XUHIZ	XUTEK	0.0032	0.0156	0.0204	0.0321	0.0453
XUSIN	XUTEK	0.0089	0.0173	0.0257	0.0336	0.0405

**Table 5:** Transfer Entropy values for k=2.

Sectors		TE from B to A	TE from A to B
XBANK	XELKT	0.027109	0.041873
XBANK	XUHIZ	0.024161	0.024251
XBANK	XUSIN	0.020949	0.035185
XBANK	XUTEK	0.025959	0.028063
XELKT	XUHIZ	0.055475	0.049321
XELKT	XUSIN	0.045621	0.040982
XELKT	XUTEK	0.033785	0.036209
XUHIZ	XUSIN	0.050213	0.048173
XUHIZ	XUTEK	0.039217	0.037592
XUSIN	XUTEK	0.044573	0.042019

**Table 6:** *p*-values of Granger causality test for k=3.

Sectors		Lag 1	Lag 2	Lag 3	Lag 4	Lag 5
XBANK	XELKT	0.7124	0.2966	0.5715	0.6823	0.8154
XBANK	XUHIZ	0.7228	0.0001	0.0003	0.0003	0.0006
XBANK	XUSIN	0.6100	0.3681	0.4677	0.5833	0.5900
XBANK	XUTEK	0.8906	0.1016	0.1913	0.1083	0.1514
XELKT	XUHIZ	0.0000	0.0000	0.0012	0.0087	0.0189
XELKT	XUSIN	0.0031	0.0172	0.0488	0.0592	0.0724
XELKT	XUTEK	0.0073	0.0208	0.0316	0.0445	0.0583
XUHIZ	XUSIN	0.0002	0.0027	0.0064	0.0089	0.0115
XUHIZ	XUTEK	0.0025	0.0143	0.0198	0.0275	0.0382
XUSIN	XUTEK	0.0091	0.0167	0.0242	0.0315	0.0410

**Table 7:** Transfer Entropy values for k=3.

Sectors		TE from B to A	TE from A to B
XBANK	XELKT	0.028504	0.021222
XBANK	XUHIZ	0.013092	0.011177
XBANK	XUSIN	0.012648	0.019466
XBANK	XUTEK	0.019415	0.014727
XELKT	XUHIZ	0.046087	0.067199
XELKT	XUSIN	0.035876	0.040012
XELKT	XUTEK	0.029157	0.031485
XUHIZ	XUSIN	0.043762	0.038213
XUHIZ	XUTEK	0.031209	0.027981
XUSIN	XUTEK	0.036528	0.034890

## Data Availability

The original contributions presented in the study are included in the article. Further inquiries can be directed to the corresponding authors.

## Appendix A. Descriptive Statistics

**Table A1:** Descriptive statistics for XUSIN.

	2022						2023						2024					
Ticker	Min	Mean	Max	Std. Dev.	Kurtosis	Skewness	Min	Mean	Max	Std. Dev.	Kurtosis	Skewness	Min	Mean	Max	Std. Dev.	Kurtosis	Skewness
ACSEL	−0.1051	0.0057	0.0953	0.0386	0.4253	0.6692	−0.1051	0.0013	0.0953	0.0412	0.4406	0.3575	−0.0881	0.0008	0.0953	0.0321	1.2862	0.5324
ADEL	−0.1049	0.0049	0.0977	0.034	1.6313	0.8235	−0.1054	0.0044	0.0959	0.0474	−0.0985	0.2968	−0.1053	0.0006	0.0951	0.0387	0.7142	0.0813
AEFES	−0.1052	0.0034	0.0954	0.0262	1.8793	0.15	−0.1051	0.003	0.0944	0.0321	0.8008	−0.0355	−0.0637	0.0022	0.095	0.0266	0.4933	0.2713
AFYON	−0.1028	0.0035	0.0939	0.0264	2.276	0.4621	−0.1057	0.0011	0.0956	0.0405	0.508	0.2885	−0.0759	0.0012	0.0952	0.0264	1.7029	0.5824
AKCNS	−0.1044	0.0048	0.0955	0.0277	2.4081	0.0228	−0.1054	0.0034	0.0952	0.0399	0.0249	0.1518	−0.0484	0.001	0.0951	0.023	1.6507	0.8241
AKSA	−0.0867	0.0038	0.0953	0.0273	0.9812	0.406	−0.0815	0.0006	0.096	0.0329	0.5805	0.382	−0.0906	0.0013	0.0947	0.0256	1.3907	0.2657
ALKA	−0.1052	0.0084	0.096	0.0377	0.5386	0.573	−0.1054	−0.0013	0.0953	0.0376	0.5163	0.1451	−0.1049	0.0011	0.0953	0.0322	1.4995	0.2532
ALKIM	−0.0734	0.0034	0.0951	0.0261	1.8248	0.4629	−0.1042	−0.0004	0.0948	0.0293	0.4404	−0.0474	−0.0866	0.0003	0.095	0.0235	2.0705	0.2609
ALMAD	−0.0998	0.0056	0.0953	0.0329	0.7615	0.504	−0.1054	0.0007	0.0953	0.0378	0.9547	0.2685	−0.0829	0.0002	0.0953	0.0283	1.3658	0.6386
ANGEN	−0.1054	0.0018	0.0946	0.0298	0.7374	0.3754	−0.1054	−0.002	0.0953	0.0381	0.4028	−0.0181	−0.1054	0.0001	0.0953	0.0357	2.108	0.5094
ARCLK	−0.0716	0.0033	0.0951	0.024	2.3956	0.6279	−0.1017	0.0006	0.095	0.033	0.3221	0.2795	−0.0596	0.0004	0.0819	0.0226	0.6768	0.0854
ARSAN	−0.1053	0.0047	0.0949	0.0296	2.7206	0.2421	−0.1054	−0.0013	0.0953	0.0439	0.2156	0.2627	−0.1054	0.0033	0.0953	0.0312	1.1531	0.35
ASUZU	−0.1055	0.0075	0.0954	0.0469	0.018	0.0276	−0.1054	−0.0006	0.0953	0.0395	0.4105	0.2259	−0.0819	0.0001	0.0952	0.0291	1.7635	0.5365
ATEKS	−0.1051	0.0037	0.0953	0.0301	1.5323	0.4207	−0.1052	−0.0009	0.095	0.037	0.2872	−0.1255	−0.1054	0.0028	0.0952	0.0352	0.8701	0.2581
AVOD	−0.104	0.0015	0.0953	0.0326	1.6779	0.0215	−0.104	−0.0004	0.0945	0.036	0.8775	0.2197	−0.0768	−0.0004	0.0941	0.0233	2.1934	0.2684
AYGAZ	−0.0966	0.0055	0.095	0.027	1.3897	−0.0404	−0.1037	0.0014	0.0952	0.0343	0.6202	0.1082	−0.1017	0.001	0.078	0.0222	1.9957	−0.0416
BAGFS	−0.0949	0.0033	0.095	0.032	1.0124	0.2072	−0.1052	−0.0008	0.0953	0.0383	0.2038	0.0794	−0.105	−0.0017	0.0947	0.0284	1.2272	−0.1938
BAKAB	−0.1052	0.0046	0.0953	0.032	1.1546	0.3275	−0.1052	0.0008	0.0953	0.0367	0.4529	−0.149	−0.1053	0.0	0.0953	0.0324	1.6269	0.6417
BANVT	−0.0855	−0.0002	0.0951	0.0191	5.4404	0.8948	−0.093	−0.0001	0.0953	0.0326	1.7673	0.3798	−0.1052	0.005	0.0953	0.0403	0.1839	0.5019
BLCYT	−0.1051	0.0023	0.0949	0.0278	2.7853	0.6695	−0.1051	−0.0012	0.0954	0.0341	0.052	−0.1417	−0.1054	0.0002	0.0946	0.0307	1.7641	−0.2876
BMSCH	−0.1049	0.0028	0.0956	0.0328	1.8649	0.6294	−0.1054	−0.0	0.0951	0.0351	1.4184	−0.1601	−0.1051	0.0034	0.0952	0.0361	1.0526	0.0653
BNTAS	−0.1034	0.0038	0.0953	0.0282	2.272	0.5677	−0.105	−0.0008	0.0946	0.0341	0.5844	−0.0769	−0.1054	0.0023	0.0953	0.0384	0.953	0.419
BOBET	−0.1067	0.0052	0.0958	0.0293	2.1862	0.4262	−0.1055	0.0029	0.0956	0.0366	0.3826	0.1095	−0.0968	−0.0012	0.0949	0.0274	0.86	0.0729
BOSSA	−0.098	0.0034	0.0955	0.0312	1.5191	0.6988	−0.1009	0.0005	0.0953	0.0314	1.2475	0.3228	−0.1054	0.0019	0.0953	0.0333	1.109	0.1406
BRISA	−0.1047	0.0029	0.0715	0.0248	0.8904	−0.2616	−0.0975	0.0013	0.0949	0.033	0.7468	0.1728	−0.1043	0.0006	0.0949	0.0287	1.4904	0.2887
BRKSN	−0.0893	0.0043	0.0953	0.0321	1.6384	0.9935	−0.1052	0.0027	0.0953	0.0474	−0.2817	0.3205	−0.1051	0.0001	0.0953	0.0358	0.8908	−0.0042
BRSAN	−0.1046	0.0044	0.0952	0.0298	1.9472	0.4104	−0.1053	0.0079	0.0953	0.0539	−0.6317	0.1102	−0.0867	−0.0013	0.0952	0.0309	1.7381	0.8442
BSOKE	−0.1049	0.0039	0.095	0.0259	2.7287	0.4518	−0.1054	0.0028	0.0953	0.0405	0.7606	0.0109	−1.3329	0.0004	0.0953	0.0942	166.6142	−11.7673
BTCIM	−0.1054	0.0041	0.0953	0.0294	2.3032	0.0637	−0.1054	0.0041	0.0953	0.039	1.1917	−0.114	−0.1054	0.0006	0.0905	0.0287	1.67	−0.2043
BUCIM	−0.1047	0.004	0.0958	0.0271	2.8545	0.1713	−0.105	0.0005	0.0955	0.0359	1.1735	0.1336	−0.0627	0.0007	0.0946	0.0215	2.414	0.4148
BURCE	−0.1053	0.0022	0.0953	0.0329	1.626	0.3356	−0.1053	0.0035	0.0953	0.0543	−0.6234	0.1826	−0.1052	0.0014	0.0953	0.0382	0.5718	0.4296
BURVA	−0.1052	0.0022	0.0953	0.0269	2.292	0.6179	−0.1054	0.0022	0.0953	0.0509	−0.3933	0.1666	−0.0926	0.0017	0.0953	0.0427	−0.073	0.4731
CCOLA	−0.0898	0.0033	0.0727	0.0226	1.1628	0.06	−0.1052	0.0039	0.0947	0.0278	1.8996	−0.0403	−0.0731	0.0009	0.0684	0.0239	0.8312	−0.1275
CELHA	−0.6178	0.0033	0.0954	0.0528	76.2376	−6.3257	−0.1045	0.0007	0.0954	0.0362	0.7645	0.3848	−0.1053	−0.0001	0.0952	0.0381	0.833	0.4915
CEMAS	−0.1044	0.0031	0.094	0.0289	1.0466	0.1005	−0.1054	0.0022	0.0951	0.0397	0.738	0.1638	−0.1054	−0.0013	0.0953	0.0358	1.9678	0.1332
CIMSA	−0.1058	0.0047	0.0876	0.0278	1.4673	0.1285	−0.1047	0.003	0.0956	0.0407	0.0976	0.4404	−0.0581	0.0018	0.0953	0.0247	1.0252	0.4774
CUSAN	−0.1043	0.0036	0.0948	0.0301	1.8579	−0.1837	−0.1051	−0.0004	0.0953	0.0398	0.3367	0.0067	−0.1054	0.0013	0.0952	0.0301	2.5148	0.1815
DAGI	−0.105	0.0037	0.0953	0.0296	1.4898	0.3331	−0.1054	0.0002	0.0953	0.0363	0.9893	0.1105	−0.1044	0.0026	0.0946	0.0314	1.264	0.0827
DARDL	−0.105	0.0027	0.0953	0.03	1.6676	0.0868	−0.1046	−0.0006	0.0948	0.0318	0.8728	0.1925	−0.1052	0.0005	0.0951	0.0343	1.4459	0.2199
DERIM	−0.1051	0.0036	0.0953	0.0293	0.7452	0.1442	−0.1054	0.0015	0.0953	0.0433	0.3308	0.1768	−0.1051	0.0032	0.0953	0.0419	0.3411	0.216
DESA	−0.105	0.0078	0.0952	0.0398	0.2042	0.2981	−0.1049	0.0015	0.0953	0.0368	0.5872	0.4167	−0.1001	0.0007	0.0953	0.0295	1.4477	0.0075
DEVA	−0.1053	0.0035	0.0778	0.0269	1.5317	−0.2897	−0.105	0.0004	0.0949	0.0339	0.7418	−0.0689	−0.1051	−0.0	0.095	0.025	2.2751	0.1213
DGNMO	−0.1051	0.0056	0.0947	0.0292	1.9394	0.0447	−0.1053	0.0004	0.0946	0.0392	0.0258	−0.0801	−0.0813	−0.0	0.0777	0.0283	0.1862	−0.2679
DITAS	−0.7079	0.0007	0.0953	0.0547	112.9278	−8.639	−0.1052	−0.0013	0.0953	0.0371	0.7888	0.0752	−0.1054	0.0001	0.0953	0.0363	1.0697	0.3702
DMSAS	−0.59	0.0006	0.0952	0.0516	68.599	−6.0166	−0.1049	−0.0011	0.0951	0.0315	1.0223	−0.1496	−0.1052	0.0021	0.0953	0.0361	2.2318	−0.2705
DNISI	−0.3894	0.0022	0.0953	0.0373	48.0256	−4.4767	−0.1056	0.0024	0.0965	0.0356	0.0853	0.1419	−0.1048	0.0032	0.0953	0.0419	0.1223	0.4169
DOGUB	−0.1054	0.0037	0.0953	0.0403	1.1075	0.2821	−0.1054	0.0013	0.0953	0.0417	0.6543	−0.1943	−0.0939	0.0016	0.0952	0.0383	0.5747	0.8019
DOKTA	−0.6865	0.001	0.0953	0.0574	82.0302	−6.6736	−0.1052	0.0001	0.0953	0.0374	0.4808	0.3269	−0.1051	−0.0006	0.0951	0.0275	1.9666	0.023
DURDO	−0.1053	0.0031	0.0953	0.0343	0.7989	0.3005	−0.1054	0.0006	0.0954	0.0374	0.5894	0.0587	−0.1054	0.0006	0.0953	0.0433	0.7954	0.1918
DYOBY	−0.1051	0.0044	0.0953	0.0313	1.4474	0.282	−0.1053	−0.0003	0.0954	0.0423	0.362	−0.0672	−0.0903	0.0041	0.0953	0.0337	0.7687	0.6788
EGGUB	−0.105	0.0024	0.0954	0.0283	1.9939	0.5312	−0.1054	0.0003	0.0951	0.0362	1.104	0.1409	−0.105	0.0008	0.0953	0.0317	1.8162	0.5787
EGPRO	−0.1049	0.0027	0.0951	0.0255	1.961	0.0972	−0.1051	0.0032	0.0953	0.0428	0.0311	0.2977	−0.0998	0.001	0.0953	0.0298	1.849	0.1838
EGSER	−0.4957	0.0026	0.0952	0.0437	67.5389	−5.9709	−0.1048	−0.0016	0.0954	0.0351	0.895	0.2872	−0.0903	0.0002	0.0943	0.0245	2.0492	0.2856
ELITE	−0.1052	0.0023	0.0955	0.037	1.3357	0.1327	−0.1054	0.0045	0.0956	0.0463	−0.2238	0.2673	−0.1051	−0.0011	0.0953	0.0359	0.9049	0.4276
EMKEL	−0.6908	0.0004	0.0956	0.0535	111.9992	−8.6769	−0.1054	0.0069	0.0954	0.058	−0.802	0.0042	−0.0981	−0.001	0.0953	0.0316	1.4348	0.8072
EPLAS	−0.1046	0.0024	0.0955	0.0341	1.5346	0.5045	−0.1053	−0.0005	0.0953	0.0437	0.2068	−0.0462	−0.0632	0.0003	0.0951	0.0264	0.7462	0.5219
ERBOS	−0.1054	0.0019	0.0951	0.0298	2.1088	0.4658	−0.1054	−0.0002	0.0953	0.0392	0.4835	0.1581	−0.0991	0.0003	0.095	0.0287	1.5394	0.357
EREGL	−0.0909	0.0019	0.0951	0.0274	1.8139	0.5635	−0.0927	−0.0	0.0949	0.0288	1.2768	0.3575	−0.0942	0.0009	0.0678	0.02	1.9214	−0.1819
ERSU	−0.1054	0.0001	0.0953	0.0273	3.7482	−0.4305	−0.1052	0.0025	0.0953	0.0342	0.8329	0.3087	−0.1054	0.002	0.0953	0.0548	−0.482	0.1425
FADE	−0.1052	0.0026	0.0953	0.0258	2.6452	0.2905	−0.1052	0.0005	0.0952	0.0366	0.466	0.1198	−0.1048	0.0004	0.0953	0.0296	2.5203	0.4144
FMIZP	−0.1052	0.0023	0.0949	0.0253	2.2614	0.4262	−0.1051	0.0014	0.0953	0.0417	0.436	0.2106	−0.0896	0.0008	0.0953	0.0323	1.0231	0.3766
FRIGO	−0.1043	0.0034	0.0952	0.0321	0.7796	0.5694	−0.0982	−0.0014	0.0953	0.0344	0.3149	−0.077	−0.1035	0.0011	0.0953	0.029	1.7671	0.1172
GEDZA	−0.1053	0.003	0.0952	0.03	1.9539	0.1468	−0.1052	0.0004	0.0953	0.038	0.3001	0.1299	−0.077	0.0008	0.0951	0.0284	0.8796	0.0755
GENTS	−0.1049	0.0026	0.0949	0.0262	2.0472	0.3122	−0.1051	0.0015	0.0948	0.0329	0.7932	0.2525	−0.066	0.0009	0.0951	0.0247	1.4044	0.4892
GEREL	−0.1031	0.0027	0.0953	0.0285	2.2692	0.4997	−0.1054	0.0036	0.0953	0.0491	−0.4307	0.1894	−0.1053	0.0011	0.0954	0.0386	1.1045	0.4259
GOLTS	−0.1051	0.0043	0.095	0.0319	1.1445	0.0362	−0.1053	0.0028	0.0952	0.0386	0.3275	0.0577	−0.1004	0.0023	0.0953	0.0345	0.9737	0.2607
GOODY	−0.1044	0.0036	0.0945	0.0254	2.2358	0.2053	−0.1053	−0.0009	0.0952	0.0348	0.4879	0.0334	−0.0925	−0.0004	0.0948	0.025	1.574	0.1212
GUBRF	−0.1053	0.0049	0.0953	0.0295	1.6414	0.4401	−0.1054	−0.0022	0.0953	0.042	0.9151	−0.3852	−0.0755	0.0025	0.0953	0.0302	1.7991	0.893
HATEK	−0.1054	0.0031	0.0954	0.0292	1.4979	0.2818	−0.105	−0.0006	0.0953	0.0362	0.5893	−0.0067	−0.1048	0.0006	0.0949	0.0322	1.164	−0.1591
HEKTS	−1.0251	0.0038	0.0953	0.0759	135.8644	−10.0158	−0.1051	−0.0025	0.0953	0.0353	0.7061	0.2829	−0.0791	−0.0027	0.0947	0.0263	2.8332	0.6904
IHEVA	−0.1027	0.0026	0.0934	0.0281	2.412	0.4699	−0.1017	0.0004	0.095	0.0382	0.524	0.1091	−0.1054	0.0012	0.0953	0.042	0.5289	0.1398
IPEKE	−0.1054	0.0037	0.0953	0.0327	1.5621	0.185	−0.105	−0.0004	0.0952	0.0398	0.1352	0.046	−0.1047	0.0018	0.0953	0.0305	1.237	0.1768
ISDMR	−0.0731	0.0017	0.0952	0.0257	2.0847	0.7898	−0.1051	0.0002	0.0953	0.034	1.4274	0.4902	−0.0837	0.0008	0.095	0.0201	2.3338	0.3358
ISKPL	−0.1033	0.002	0.0943	0.027	2.7898	0.3477	−0.1054	−0.0004	0.0953	0.0348	1.1746	−0.2877	−0.0914	0.0039	0.0803	0.0269	1.2051	−0.4006
ISSEN	−0.1033	0.0073	0.0953	0.0223	4.7693	0.4223	−0.1055	−0.0041	0.0955	0.0351	1.1854	−0.3943	−0.1004	−0.0005	0.0959	0.0232	2.9078	0.3492
IZFAS	−0.0767	0.0026	0.0953	0.0294	1.682	0.775	−0.1054	0.0037	0.0953	0.0367	0.5941	−0.2122	−0.1053	0.0062	0.0952	0.0316	3.739	−0.4194
IZINV	−0.1052	0.0009	0.095	0.0285	2.0139	0.735	−0.1053	0.0016	0.0953	0.0404	0.4457	0.2201	−0.1049	0.0008	0.0953	0.0371	0.9264	0.3277
IZMDC	−0.1013	0.0035	0.0938	0.0279	1.9865	0.268	−0.1054	0.0001	0.0953	0.0362	1.0721	0.1909	−0.0798	−0.0001	0.0953	0.0243	1.8551	0.358
JANTS	−0.105	0.0032	0.0953	0.0291	2.5428	0.7005	−0.0948	−0.0003	0.0952	0.0352	0.5865	0.2063	−0.1052	0.002	0.0953	0.0402	1.0425	0.5518
KAPLM	−0.1054	0.0058	0.0953	0.0561	−0.7102	−0.0318	−0.1054	−0.0012	0.0953	0.0397	0.7763	0.3781	−0.1054	0.0034	0.0953	0.0426	0.5004	0.5237
KARSN	−0.1054	0.0049	0.0953	0.0335	0.887	0.3663	−0.1052	−0.0015	0.0948	0.0345	0.5108	−0.0503	−0.1052	0.0015	0.0953	0.0364	1.6741	0.4403
KARTN	−0.1051	0.0025	0.095	0.0276	1.6929	0.0943	−0.0944	−0.0012	0.0952	0.0301	0.6589	0.0663	−0.1046	0.0008	0.0953	0.0372	1.3146	0.4148
KATMR	−0.0981	0.0028	0.0937	0.0268	1.811	0.1516	−0.1054	−0.0011	0.0944	0.039	0.8291	0.0422	−0.1051	0.0007	0.095	0.0339	1.4738	0.3756
KERVT	−0.1035	0.0051	0.0948	0.0321	0.9869	−0.105	−0.1007	−0.0025	0.0762	0.0326	0.1015	−0.4246	−0.104	0.0018	0.0951	0.0311	0.6708	0.0076
KLKIM	−0.1066	0.0039	0.0959	0.0318	1.128	0.3628	−0.1043	0.0023	0.0957	0.0413	−0.0198	0.3027	−0.1048	0.0015	0.095	0.0274	1.134	0.4037
KLMSN	−0.1054	0.0032	0.0953	0.031	2.2883	0.1902	−0.1019	−0.0002	0.0953	0.0363	0.3906	0.1304	−0.1053	0.001	0.0951	0.0305	1.5335	−0.2633
KNFRT	−0.1054	0.0048	0.0953	0.034	1.1576	0.3425	−0.1055	0.0008	0.0958	0.0421	0.2679	0.0329	−0.1053	0.0008	0.0946	0.0311	0.9421	−0.1575
KONKA	−0.105	0.0086	0.0957	0.0408	0.0522	0.3622	−0.1054	−0.0012	0.0953	0.043	0.2154	0.189	−0.1037	−0.0006	0.0953	0.0284	2.5014	0.0063
KORDS	−0.1055	0.0042	0.0952	0.0292	0.9816	0.2233	−0.0881	−0.0009	0.0949	0.0313	0.876	0.2016	−0.0953	−0.0003	0.0946	0.0247	2.1273	−0.1344
KOZAA	−0.1052	0.0042	0.0953	0.0347	1.2464	0.1301	−0.1046	−0.0009	0.0952	0.0394	−0.0396	0.1672	−0.0857	0.0018	0.0947	0.0279	0.767	−0.1539
KOZAL	−0.105	0.0065	0.0958	0.0356	1.1568	0.2002	−0.0971	−0.0012	0.0954	0.0368	0.2691	0.1913	−0.0997	0.0005	0.0953	0.0271	1.9036	−0.029
KRDMA	−0.105	0.0037	0.0954	0.033	1.4351	0.4845	−0.1053	0.0005	0.0953	0.0383	0.6782	0.2142	−0.0771	0.0014	0.0953	0.0254	1.3397	0.3309
KRDMB	−0.1051	0.0033	0.0953	0.0322	1.8784	0.5047	−0.1051	0.0006	0.0953	0.0355	1.3087	0.1987	−0.0711	0.001	0.095	0.0241	1.5038	0.4557
KRDMD	−0.1052	0.0028	0.0953	0.0336	0.8003	0.3132	−0.105	0.0012	0.0954	0.0376	0.4356	0.2749	−0.0891	0.0007	0.0951	0.0249	0.8538	−0.0118
KRSTL	−0.1054	0.0048	0.0953	0.0449	−0.0162	0.0095	−0.1054	0.0007	0.0953	0.0469	−0.1894	0.0928	−0.1052	−0.0028	0.0953	0.0305	2.4803	0.5483
KRTEK	−0.0878	0.0051	0.0953	0.0334	0.7427	0.5703	−0.1054	−0.0004	0.0953	0.036	0.7921	−0.0839	−0.105	0.0015	0.0952	0.0328	0.9133	0.2001
KRVGD	−0.1082	0.0034	0.0941	0.0264	2.8516	0.1896	−0.1003	−0.0014	0.099	0.0322	1.1803	0.1558	−0.1035	0.0001	0.0658	0.0258	1.3038	−0.4835
KTSKR	−0.1047	0.0041	0.0951	0.0313	0.8537	0.3236	−0.1054	−0.0009	0.0954	0.039	0.6361	0.0426	−0.1052	−0.0003	0.0952	0.0274	2.361	0.0488
KUTPO	−0.1054	−0.0026	0.095	0.0322	2.6112	−0.5061	−0.1053	−0.0006	0.095	0.0335	1.28	0.0124	−0.0934	0.0014	0.0953	0.0319	0.7498	0.1961
LUKSK	−0.1045	0.0023	0.0953	0.0307	1.9588	0.8266	−0.1053	0.0027	0.0953	0.0466	−0.0675	0.195	−0.0891	0.0015	0.0953	0.0362	0.6271	0.5161
MAKTK	−0.1019	0.0032	0.0946	0.0255	3.0055	−0.0163	−0.1043	−0.0013	0.0951	0.0348	0.6827	−0.0285	−0.1054	0.0012	0.0951	0.0359	1.3149	0.2756
MEDTR	−0.1048	0.0044	0.0954	0.0295	1.16	0.2262	−0.1054	−0.0013	0.0953	0.0364	0.405	0.2381	−0.0745	0.0019	0.0952	0.0255	0.8582	0.1237
MERCN	−0.1049	0.0059	0.0959	0.0325	0.9638	0.2342	−0.1057	−0.0002	0.0956	0.0414	0.129	0.0497	−0.0784	−0.0006	0.0953	0.0256	1.0649	0.0972
MERKO	−0.1054	0.0027	0.0953	0.0326	1.8167	0.7124	−0.1051	−0.0003	0.0951	0.038	0.4722	0.0705	−0.0835	0.0022	0.0953	0.0351	0.5738	0.5718
MNDRS	−0.1054	0.0055	0.0953	0.0375	0.9169	−0.1531	−0.1054	−0.0001	0.0953	0.0423	0.0332	0.1068	−0.1051	0.0006	0.0953	0.0303	2.0746	−0.2171
MNDTR	−0.1051	0.0033	0.0954	0.0347	0.9458	0.3952	−0.1002	−0.0018	0.0949	0.03	0.1739	0.0376	−0.0943	0.0001	0.0953	0.0268	1.6146	0.1965
NIBAS	−0.1045	0.0007	0.0952	0.0313	2.2688	0.8574	−0.1054	0.0001	0.0953	0.0396	0.6131	0.1964	−0.1051	0.0004	0.0953	0.0343	1.9157	0.6906
NUHCM	−0.1051	0.0035	0.0949	0.0284	1.6403	−0.1138	−0.1054	0.004	0.0953	0.0446	−0.0113	0.281	−0.0882	−0.0001	0.0953	0.0289	1.7674	0.543
OTKAR	−0.1053	0.0042	0.0953	0.0255	2.9068	0.5963	−0.1053	0.0029	0.0953	0.0332	0.9788	−0.1915	−0.0691	0.0004	0.0953	0.0239	1.4704	0.3516
OYAKC	−0.1047	0.0037	0.0931	0.0296	0.7569	0.0849	−0.096	0.0039	0.0953	0.0407	−0.0914	0.4111	−0.0704	0.0019	0.0947	0.0246	0.5804	0.1042
OYLUM	−0.1036	0.0029	0.0951	0.0343	0.9922	0.7045	−0.1054	0.0016	0.0953	0.0402	0.4069	0.1617	−0.1049	0.0008	0.0951	0.0328	1.4619	0.0789
OZRDN	−0.1053	0.0028	0.0952	0.0331	1.7552	0.4471	−0.1054	0.004	0.0951	0.0401	0.1728	−0.0088	−0.1048	−0.0013	0.0952	0.033	1.7264	0.0865
PARSN	−0.1049	0.0034	0.0953	0.0301	1.3682	0.2254	−0.1053	0.0006	0.0951	0.0392	0.2622	0.0352	−0.1048	−0.0	0.095	0.0283	1.3949	0.1764
PENGD	−0.1044	0.0037	0.0952	0.0305	1.4288	0.2875	−0.1047	−0.0019	0.095	0.0356	0.4279	−0.254	−0.1052	0.0006	0.0946	0.0277	2.1733	−0.0784
PETKM	−0.105	0.0036	0.0952	0.0305	1.8862	−0.2395	−0.1049	−0.0004	0.0953	0.0317	0.7839	−0.0287	−0.1051	0.0	0.0738	0.0244	2.2504	−0.4586
PETUN	−0.0962	0.0054	0.0966	0.0267	2.861	0.7448	−0.1006	−0.0003	0.0954	0.0346	0.8158	0.165	−0.0887	0.0005	0.0836	0.0257	1.1168	0.2185
PINSU	−0.1055	0.0033	0.0951	0.031	2.5814	−0.1601	−0.1046	0.0019	0.0952	0.0376	0.5813	0.1195	−0.0774	0.002	0.0954	0.0251	1.589	0.186
PNSUT	−0.1044	0.006	0.0953	0.0258	2.1024	0.1433	−0.1053	−0.0002	0.0953	0.0351	0.7441	−0.2554	−0.0942	0.0002	0.0948	0.0237	2.6758	0.0607
PRKAB	−0.1054	−0.0024	0.0953	0.0373	1.0303	0.6568	−0.1051	−0.0007	0.0952	0.0376	0.7142	0.1444	−0.1054	0.0	0.0953	0.0329	1.8359	0.3186
PRKME	−0.1134	0.0052	0.0953	0.0372	1.2422	0.2484	−0.1052	−0.001	0.0953	0.0388	0.4474	0.2052	−0.0838	0.0	0.0949	0.0253	2.4714	0.4672
PRZMA	−0.1035	0.0034	0.0953	0.0266	3.2896	0.8537	−0.1054	0.0065	0.0953	0.037	3.1533	−1.2719	−0.1053	−0.0029	0.0953	0.0374	0.9337	−0.1224
QUAGR	−0.0838	0.0074	0.0972	0.0302	1.0814	0.4314	−0.1049	−0.0009	0.0943	0.0314	0.991	−0.0121	−0.1046	−0.0025	0.0951	0.0254	3.8149	−0.2866
RNPOL	−0.1046	0.0022	0.0953	0.0279	2.3147	0.2249	−0.1008	0.0025	0.0953	0.0343	0.592	−0.0038	−0.0778	−0.0018	0.0953	0.0283	1.5513	0.4617
RODRG	−0.1051	0.0029	0.0953	0.0403	0.5911	0.3732	−0.1054	0.0017	0.0953	0.0523	−0.3803	0.2317	−0.1054	0.0017	0.0953	0.043	0.2127	0.4621
RTALB	−0.1052	0.0013	0.0956	0.0324	2.463	0.9664	−0.1053	0.0004	0.0953	0.0413	0.398	0.233	−0.1054	0.0001	0.0953	0.0413	1.1885	0.3603
SAFKR	−0.1042	0.0037	0.0951	0.029	1.6031	0.0314	−0.1054	0.0023	0.0953	0.0428	0.2025	0.0695	−0.105	0.0023	0.0953	0.0382	0.1972	−0.3266
SAMAT	−0.0917	0.0063	0.0949	0.0234	4.0645	0.7068	−0.1054	0.003	0.0953	0.0528	−0.0338	−0.1787	−0.1053	−0.0033	0.0953	0.0465	0.4097	0.146
SANFM	−0.1055	0.0017	0.0836	0.0316	1.1638	−0.2553	−0.1053	0.0027	0.0954	0.0343	1.1667	0.375	−0.1051	0.0079	0.0954	0.0385	0.6329	−0.2801
SARKY	−0.0891	0.0029	0.0952	0.0264	1.5741	0.5796	−0.1052	0.0005	0.0953	0.0404	0.2417	0.0713	−0.0716	−0.0011	0.0856	0.0238	0.8449	0.2087
SASA	−0.1054	0.0057	0.0952	0.0385	1.0177	−0.0563	−0.1053	−0.0011	0.0953	0.0359	0.993	0.1582	−0.0949	−0.0005	0.0952	0.0271	1.0767	0.367
SEKUR	−1.1723	−0.0026	1.1536	0.2421	19.5888	−0.5519	−0.1053	−0.0009	0.0951	0.0355	0.9574	0.0628	−0.1052	0.0027	0.0953	0.038	0.6224	0.1539
SELGD	−0.0739	0.002	0.0951	0.0275	2.08	1.0272	−0.1054	0.0045	0.0953	0.042	0.3451	0.5613	−0.1052	0.0037	0.0953	0.0426	0.0787	0.5182
SELVA	−0.1054	−0.0001	0.0953	0.0423	0.9668	−0.1654	−0.1028	−0.0008	0.0953	0.0359	0.521	0.1613	−0.105	−0.0003	0.0953	0.0325	2.2668	0.3384
SEYKM	−0.7013	−0.0008	0.0953	0.055	105.6827	−8.1692	−0.1048	−0.0007	0.0957	0.0339	0.7936	0.135	−0.1032	0.0006	0.0953	0.0344	1.6207	0.2876
SILVR	−0.1054	0.005	0.0953	0.0401	1.4375	−0.4716	−0.1052	−0.0009	0.0953	0.0332	0.9966	−0.0074	−0.1054	0.0014	0.0953	0.0363	0.8466	0.3327
SKTAS	−0.1056	0.0036	0.095	0.0333	1.3384	−0.3438	−0.1048	−0.0004	0.095	0.0415	0.3139	−0.1482	−0.1043	0.0004	0.0953	0.0359	1.1773	0.3794
TATGD	−0.0827	0.0044	0.0951	0.0282	1.0599	0.381	−0.1054	−0.0003	0.0953	0.0365	0.7407	0.2116	−0.0724	−0.0018	0.0862	0.0253	0.6108	0.3316
TBORG	−0.0905	0.0029	0.0953	0.0255	2.8941	0.5955	−0.105	0.0026	0.0953	0.0331	1.3894	0.4626	−0.1051	0.0028	0.0953	0.0307	1.8356	0.3694
TEZOL	−0.1039	0.0057	0.0949	0.0295	1.4059	0.2534	−0.0971	0.0008	0.095	0.0327	0.3171	−0.0551	−0.1016	−0.0003	0.0954	0.0291	1.2044	0.1864
TMPOL	−0.1052	0.0031	0.0952	0.0296	1.5209	0.4221	−0.1054	0.0044	0.0953	0.0449	−0.2334	0.1887	−0.1052	−0.0011	0.0953	0.0337	1.3738	0.4991
TMSN	−0.1052	0.0049	0.0952	0.0358	0.9988	0.5631	−0.1054	0.001	0.0953	0.0418	0.1839	0.3357	−0.1054	0.0012	0.0953	0.0351	1.2692	0.292
TOASO	−0.1054	0.0028	0.095	0.0285	1.9376	−0.1323	−0.1054	0.0011	0.0951	0.0323	0.5329	−0.1303	−0.0887	−0.0001	0.0653	0.0236	0.7561	0.0125
TRILC	−0.1052	0.0006	0.0951	0.0249	2.1463	0.0711	−0.1054	−0.0007	0.0953	0.0335	1.216	0.2309	−0.1054	0.0014	0.0953	0.0365	1.282	−0.0702
TUCLK	−0.1052	0.0036	0.0953	0.0332	1.2733	0.4462	−0.1054	0.0022	0.0954	0.0436	0.3262	0.1571	−0.0971	−0.0002	0.0953	0.0349	1.233	0.5138
TUKAS	−0.0958	0.0063	0.0948	0.0316	0.4316	0.6292	−0.105	−0.0004	0.0953	0.0398	0.3725	0.0442	−0.0916	0.0	0.095	0.0246	1.134	0.175
TUPRS	−0.1314	0.0041	0.0951	0.0328	1.782	−0.1087	−0.1022	0.0031	0.0953	0.0322	0.9013	0.283	−0.0363	0.0006	0.0561	0.0169	0.1381	0.5219
ULKER	−0.1054	0.0036	0.0953	0.0258	3.164	0.554	−0.1053	0.0025	0.0953	0.0412	0.4897	0.0024	−0.0918	0.0011	0.095	0.029	0.7032	0.3955
ULUSE	−0.1054	0.0055	0.0953	0.0451	0.3504	0.3818	−0.1054	−0.0023	0.0953	0.0453	0.3491	0.1992	−0.0951	0.0009	0.0953	0.033	0.7682	0.6894
ULUUN	−0.1051	0.0067	0.0954	0.0394	0.4426	0.544	−0.1051	−0.0011	0.0951	0.0382	0.3054	0.1031	−0.082	−0.0002	0.0951	0.0257	3.2229	0.7902
USAK	−0.1038	0.0042	0.0953	0.0326	1.5818	0.0674	−0.1054	0.0037	0.0953	0.0422	0.1186	0.181	−0.1054	0.0011	0.0953	0.0461	0.3832	0.3482
VANGD	−0.0455	0.0012	0.095	0.024	5.5531	1.9845	−0.1054	0.0043	0.0953	0.0571	−0.7157	−0.0008	−0.0838	0.0008	0.0953	0.0336	0.9747	0.4753
VESBE	−0.1049	0.0021	0.0949	0.022	4.2809	0.2079	−0.0943	0.0007	0.0946	0.0294	0.5968	0.1487	−0.0696	0.0004	0.0953	0.0233	2.7416	0.5003
VESTL	−0.1049	0.0039	0.095	0.0288	1.5177	0.1446	−0.1054	−0.0017	0.0951	0.0347	0.3257	0.1747	−0.0734	0.0015	0.0953	0.0327	1.3116	0.8446
VKING	−0.1054	0.0065	0.0953	0.0426	1.0527	0.3265	−0.1054	−0.0011	0.0953	0.0501	0.1413	0.2129	−0.1051	0.0015	0.0953	0.0428	0.5018	0.1756
YAPRK	−0.1054	0.002	0.0953	0.0288	3.5191	0.8266	−0.1054	0.0061	0.0953	0.0505	−0.3694	0.3797	−0.1052	0.004	0.0953	0.0404	0.5437	0.3404
YATAS	−0.1051	0.0044	0.0952	0.0275	2.6569	0.141	−0.1048	−0.0012	0.0948	0.0301	0.3261	−0.1356	−0.0893	0.0004	0.0951	0.0246	2.6099	0.4406
YKSLN	−0.1057	0.0035	0.0956	0.0279	2.8139	0.0092	−0.1061	0.0003	0.0961	0.0367	0.7863	−0.0338	−0.1049	0.0	0.0953	0.0323	1.4643	0.1186
YUNSA	−0.1061	0.0077	0.0954	0.036	0.5805	0.4549	−0.105	0.0024	0.095	0.0368	0.2743	0.1203	−0.0975	−0.0014	0.0952	0.0264	2.1641	0.3998

**Table A2:** Descriptive statistics for XELKT.

	2022						2023						2024					
Ticker	Min	Mean	Max	Std. Dev.	Kurtosis	Skewness	Min	Mean	Max	Std. Dev.	Kurtosis	Skewness	Min	Mean	Max	Std. Dev.	Kurtosis	Skewness
AKENR	−0.1048	0.0043	0.0953	0.0331	1.8423	0.4782	−0.0889	−0.0009	0.0953	0.0358	0.7272	0.3743	−0.1051	0.0039	0.0953	0.0458	0.2901	0.4296
AKSEN	−0.0938	0.0061	0.095	0.0292	0.7868	0.1484	−0.0882	−0.0017	0.0953	0.0319	0.5342	0.0704	−0.0853	0.0009	0.0651	0.0227	0.9742	−0.2066
AKSUE	−0.6453	−0.0006	0.0847	0.0471	140.7772	−10.3216	−0.1055	−0.0029	0.0847	0.0279	1.3726	−0.3339	−0.0747	0.0002	0.0953	0.027	1.8679	0.5942
ARASE	−0.0962	0.0038	0.0952	0.0303	2.4212	0.6308	−0.1051	0.0001	0.0953	0.035	1.0211	0.2184	−0.105	−0.0006	0.0952	0.028	2.4883	−0.3351
AYDEM	−0.1051	0.0058	0.0951	0.0319	1.3642	0.1406	−0.105	−0.0017	0.0953	0.0364	0.3244	−0.0091	−0.1047	0.0013	0.0951	0.0276	1.3005	−0.2055
AYEN	−0.1053	0.0063	0.0953	0.0372	0.74	0.2102	−0.105	−0.0018	0.0953	0.0369	0.686	0.2718	−0.0919	0.0011	0.0953	0.0269	1.9899	0.351
BIOEN	−0.0905	0.0047	0.0952	0.0273	2.2924	0.5259	−0.1054	−0.0004	0.0951	0.0372	0.6388	0.2456	−0.0928	0.0002	0.0949	0.0269	2.0279	0.3648
CANTE	−0.1021	0.0093	0.096	0.0371	0.5706	0.5838	−0.1065	−0.0045	0.0969	0.0458	0.36	−0.2035	−0.087	−0.0011	0.0973	0.0228	2.9073	0.2017
ENJSA	−0.1045	0.0041	0.0904	0.0256	1.8532	0.134	−0.1054	0.0012	0.095	0.0335	0.93	−0.0912	−0.0667	0.0013	0.06	0.0207	0.3547	−0.1481
GWIND	−0.1055	0.0051	0.095	0.0297	1.3293	0.1977	−0.1051	0.0	0.0954	0.0353	0.5316	0.2265	−0.0728	0.0014	0.0602	0.0232	0.2887	−0.2262
KARYE	−0.1053	0.003	0.0953	0.0354	1.2035	0.178	−0.1049	−0.0014	0.0953	0.0375	0.3089	0.0026	−0.1051	0.001	0.0953	0.0416	0.5897	0.5833
MAGEN	−0.105	0.0048	0.0953	0.0325	1.4891	0.4363	−0.1062	−0.0007	0.0957	0.0381	0.7455	−0.0306	−0.0802	0.0029	0.0953	0.036	1.0943	0.6623
NATEN	−0.1056	0.0057	0.0954	0.0345	1.0656	0.6643	−0.1052	0.0007	0.0956	0.0485	−0.4056	0.1553	−0.1048	0.0008	0.0952	0.0291	2.1124	0.7365
ODAS	−0.1054	0.0083	0.0953	0.0366	0.7043	−0.1975	−0.1052	−0.0013	0.0953	0.0397	0.0355	0.229	−0.0936	−0.0011	0.07	0.0254	0.8028	−0.3251
PAMEL	−0.1054	0.0042	0.0952	0.0344	2.5469	−0.6694	−0.1054	−0.0035	0.0953	0.0421	0.6872	−0.0454	−0.097	−0.0004	0.0953	0.0283	1.8151	0.312
ZEDUR	−0.1055	0.0067	0.0953	0.0434	0.3503	0.0442	−0.1054	0.0006	0.0954	0.0471	−0.0958	0.27	−0.1053	−0.0014	0.0954	0.0362	1.5336	0.1713
ZOREN	−0.1045	0.0057	0.0953	0.0346	1.4526	0.3869	−0.1049	−0.0001	0.0953	0.0387	0.4081	0.3184	−0.1037	0.0001	0.0941	0.0262	2.6477	0.2496

**Table A3:** Descriptive statistics for XUHIZ.

	2022						2023						2024					
Ticker	Min	Mean	Max	Std. Dev.	Kurtosis	Skewness	Min	Mean	Max	Std. Dev.	Kurtosis	Skewness	Min	Mean	Max	Std. Dev.	Kurtosis	Skewness
ADESE	−0.091	0.0037	0.0925	0.0286	1.3821	0.6322	−0.105	−0.0003	0.0946	0.0351	1.5587	−0.007	−0.0812	0.0009	0.0946	0.0268	1.5668	0.5119
AKENR	−0.1048	0.0043	0.0953	0.0331	1.8423	0.4782	−0.0889	−0.0009	0.0953	0.0358	0.7272	0.3743	−0.1051	0.0039	0.0953	0.0457	0.3042	0.4324
AKSEN	−0.0938	0.0061	0.095	0.0292	0.7868	0.1484	−0.0882	−0.0017	0.0953	0.0319	0.5342	0.0704	−0.0853	0.0008	0.0651	0.0226	0.9738	−0.1993
AKSUE	−0.6453	−0.0006	0.0847	0.0471	140.7772	−10.3216	−0.1055	−0.0029	0.0847	0.0279	1.3726	−0.3339	−0.0747	0.0002	0.0953	0.0269	1.8879	0.5955
ANELE	−0.1044	0.0022	0.094	0.0285	1.7607	−0.0719	−0.104	0.0041	0.0953	0.0367	1.0	0.3207	−0.1041	0.001	0.0953	0.0354	1.1583	0.1519
ARASE	−0.0962	0.0038	0.0952	0.0303	2.4212	0.6308	−0.1051	0.0001	0.0953	0.035	1.0211	0.2184	−0.105	−0.0007	0.0952	0.0279	2.5054	−0.3321
ARZUM	−0.1048	0.0021	0.0947	0.0242	3.5036	0.3228	−0.1053	0.0009	0.0955	0.0381	0.7733	0.3655	−0.1052	0.0007	0.0953	0.0389	0.8976	0.4646
AVTUR	−0.1044	0.0012	0.0941	0.0254	2.8885	0.5575	−0.1054	0.0035	0.0953	0.05	−0.3062	0.1689	−0.1054	0.0017	0.0953	0.0449	0.1083	0.3659
AYDEM	−0.1051	0.0058	0.0951	0.0319	1.3642	0.1406	−0.105	−0.0017	0.0953	0.0364	0.3244	−0.0091	−0.1047	0.0013	0.0951	0.0275	1.316	−0.2034
AYEN	−0.1053	0.0063	0.0953	0.0372	0.74	0.2102	−0.105	−0.0018	0.0953	0.0369	0.686	0.2718	−0.0919	0.0011	0.0953	0.0268	2.0095	0.3506
BEYAZ	−0.1055	0.003	0.0954	0.0321	1.8107	0.5495	−0.1054	0.0003	0.0953	0.0471	−0.044	0.0848	−0.1048	0.0017	0.0953	0.0343	1.0902	0.2751
BIM	−0.063	0.003	0.0823	0.0234	0.6485	0.0786	−0.1052	0.0033	0.0953	0.0279	1.692	0.0429	−0.0782	0.0021	0.074	0.0236	0.7009	0.0539
BIOEN	−0.0905	0.0047	0.0952	0.0273	2.2924	0.5259	−0.1054	−0.0004	0.0951	0.0372	0.6388	0.2456	−0.0928	0.0002	0.0949	0.0269	2.0485	0.3656
BIZIM	−0.1048	0.004	0.0951	0.0287	1.6511	0.4625	−0.1051	0.0002	0.0952	0.0342	0.7094	−0.1409	−0.0633	−0.0012	0.0584	0.0229	−0.0217	−0.133
BJKAS	−1.4969	−0.0046	0.0953	0.0994	205.1163	−13.6183	−0.0985	0.008	0.0958	0.0362	0.6776	0.328	−0.1052	−0.0033	0.0953	0.0452	0.7108	−0.1393
BRLSM	−0.1071	0.0052	0.0954	0.0319	2.1988	0.7187	−0.1052	0.0022	0.0952	0.0465	−0.1636	0.1673	−0.105	−0.0017	0.0953	0.031	1.6957	0.6129
CANTE	−0.1021	0.0093	0.096	0.0371	0.5706	0.5838	−0.1065	−0.0045	0.0969	0.0458	0.36	−0.2035	−0.087	−0.0011	0.0973	0.0228	2.9301	0.2046
CEOEM	−0.1057	0.0013	0.0953	0.0349	2.2282	−0.2761	−0.1054	0.0004	0.0953	0.048	−0.1539	0.1157	−0.1054	0.002	0.0953	0.0425	0.4605	−0.3258
CRFSA	−0.1053	0.0023	0.0953	0.0298	3.5267	1.1013	−0.1052	0.0016	0.0953	0.0415	0.3121	0.2827	−0.0837	−0.0003	0.0953	0.0284	1.4761	0.6561
DOAS	−0.2255	0.0052	0.0953	0.0356	7.41	−0.8231	−0.1054	0.0016	0.0952	0.0343	1.0761	0.1593	−0.0648	−0.0004	0.0804	0.0217	0.8716	0.1284
DOBUR	−0.1054	−0.0007	0.0953	0.047	0.0142	0.3487	−0.1054	−0.0001	0.0953	0.0465	0.0379	0.2348	−0.1051	0.004	0.0953	0.0478	−0.1321	0.2041
EDIP	−0.1046	0.0056	0.0953	0.0355	1.0915	0.6465	−0.1054	0.0006	0.0953	0.0563	−0.6005	0.1091	−0.1049	0.0017	0.0953	0.0361	1.1764	0.5085
EGEPO	−0.1039	0.0033	0.0953	0.0293	2.5197	0.3245	−0.1054	0.0004	0.0951	0.0391	0.8661	−0.4452	−0.1048	0.0007	0.0953	0.0305	0.8088	−0.0896
ENJSA	−0.1045	0.0041	0.0904	0.0256	1.8532	0.134	−0.1054	0.0012	0.095	0.0335	0.93	−0.0912	−0.0667	0.0013	0.06	0.0207	0.3676	−0.1466
ENKAI	−0.1047	0.0033	0.0949	0.0293	1.7698	0.1654	−0.1051	0.0001	0.0952	0.0315	0.9733	−0.1361	−0.0507	0.0019	0.0841	0.0221	0.3073	0.3079
ESCAR	−0.1049	0.0046	0.0967	0.0309	1.812	0.5226	−0.105	0.0021	0.0956	0.033	1.5537	0.3804	−0.0707	0.0039	0.0953	0.028	1.4146	0.7228
ESEN	−0.1063	0.0033	0.0959	0.0308	2.0314	0.4974	−0.1051	−0.0006	0.0953	0.0383	0.2772	0.4166	−0.0656	0.0014	0.0946	0.0267	1.3437	0.4907
ETILR	−0.1055	0.0033	0.0952	0.0382	0.6659	0.5289	−0.1054	−0.0004	0.0953	0.0362	1.2246	−0.1565	−0.1052	0.0028	0.0954	0.0486	−0.1184	0.0019
FENER	−0.1049	0.0037	0.0953	0.0328	1.5715	0.0493	−0.1054	0.0009	0.0953	0.0389	1.014	−0.2098	−0.8454	−0.0034	0.0953	0.0667	104.484	−8.2974
FLAP	−0.1014	0.0066	0.0953	0.0376	0.4355	0.6221	−0.1053	−0.0004	0.0954	0.0504	−0.2379	0.0765	−0.1054	−0.0008	0.0954	0.0422	0.6002	0.1154
GENIL	−0.105	0.0049	0.0952	0.0268	2.2853	0.0469	−0.1052	−0.0003	0.095	0.0352	0.423	0.0797	−0.0695	0.0036	0.0788	0.0244	0.6789	0.1469
GESAN	−0.1058	0.0103	0.0961	0.0422	0.3543	0.044	−0.1054	0.0005	0.0953	0.048	−0.3637	0.0545	−0.0824	−0.0014	0.0817	0.0275	0.5434	0.5217
GMTAS	−0.1061	0.0042	0.0942	0.0263	2.9573	0.0436	−0.1044	0.0009	0.0955	0.0355	0.8822	0.2645	−0.0994	0.0015	0.0953	0.0287	1.2845	0.401
GSDDE	−0.1054	0.0037	0.0948	0.0315	1.0637	−0.0508	−0.1054	−0.0009	0.0944	0.0358	0.3353	−0.1007	−0.1054	0.0008	0.0953	0.0331	2.255	0.2915
GSRAY	−0.1068	0.0034	0.0976	0.031	2.0956	0.5404	−0.1054	0.0015	0.0965	0.0405	0.5451	−0.071	−0.1052	−0.0001	0.0952	0.0306	1.7875	0.2429
GWIND	−0.1055	0.0051	0.095	0.0297	1.3293	0.1977	−0.1051	0.0	0.0954	0.0353	0.5316	0.2265	−0.0728	0.0014	0.0602	0.0231	0.3011	−0.2248
HURGZ	−0.1039	0.0049	0.0949	0.0346	1.2018	0.4117	−0.1054	−0.0019	0.0953	0.0397	0.4883	0.0758	−0.1049	0.0008	0.0949	0.0288	2.1406	−0.0375
IHAAS	−0.1051	0.0088	0.0969	0.0442	−0.1906	0.3727	−0.1054	−0.0036	0.0955	0.0483	0.2801	−0.1748	−0.1054	0.0009	0.0953	0.0411	1.4154	−0.3873
IHGZT	−0.1035	0.0025	0.0941	0.031	2.4216	0.5609	−0.1024	−0.0005	0.0935	0.0362	0.2197	−0.0315	−0.1037	0.0013	0.0947	0.0391	0.9025	0.3873
IHLGM	−0.0994	0.0017	0.0945	0.0277	2.5005	0.3842	−0.1054	0.0002	0.0953	0.039	0.2878	0.0019	−0.1054	0.0024	0.0953	0.0397	0.8061	0.2091
INTEM	−0.1054	0.0001	0.0953	0.0413	0.4903	0.1357	−0.1054	0.0029	0.0953	0.0464	−0.0345	0.3486	−0.1051	0.0005	0.0953	0.0352	0.9198	0.4168
KARYE	−0.1053	0.003	0.0953	0.0354	1.2035	0.178	−0.1049	−0.0014	0.0953	0.0375	0.3089	0.0026	−0.1051	0.001	0.0953	0.0416	0.5938	0.5784
KIMMR	−0.1054	0.0038	0.0953	0.039	2.1647	−0.4016	−0.1054	−0.0012	0.0951	0.0367	0.7719	0.092	−0.1045	0.0018	0.0953	0.0366	1.2426	0.6044
KONTR	−0.1052	0.01	0.0954	0.0422	0.445	0.1772	−0.1051	0.0013	0.0954	0.0439	−0.1533	0.349	−0.1055	−0.0022	0.0953	0.0325	1.5209	0.4932
KUYAS	−0.1047	0.0062	0.0952	0.0345	1.1621	0.2627	−0.1054	0.0028	0.0953	0.0406	0.94	−0.161	−0.074	0.0014	0.0953	0.0299	1.1745	0.6446
LKMNH	−0.1049	0.004	0.0951	0.0277	1.5094	0.5032	−0.1054	0.0026	0.0953	0.0312	1.4808	−0.0	−0.1048	0.0019	0.0953	0.0299	2.4448	−0.526
MAGEN	−0.105	0.0048	0.0953	0.0325	1.4891	0.4363	−0.1062	−0.0007	0.0957	0.0381	0.7455	−0.0306	−0.0802	0.003	0.0953	0.036	1.0832	0.6534
MARTI	−0.1054	0.0077	0.0947	0.0309	3.1627	−0.4486	−0.1054	−0.003	0.0953	0.0406	0.8554	−0.0714	−0.1044	−0.0003	0.0953	0.0309	2.1372	−0.274
MAVI	−0.1052	0.0053	0.0952	0.0235	2.0745	−0.1352	−0.1051	0.0024	0.0949	0.0311	0.7243	−0.1315	−0.071	0.0017	0.0911	0.025	0.8254	0.4447
MEPET	−0.1054	0.0039	0.0953	0.0377	1.1047	0.3531	−0.1044	0.0008	0.0952	0.0407	0.3775	0.115	−0.1054	0.0008	0.0953	0.0366	0.9103	0.3253
MERIT	−0.1054	0.0012	0.0952	0.0299	2.3423	0.394	−0.0992	0.002	0.0955	0.0391	0.4161	0.2861	−0.101	0.0041	0.0953	0.0416	−0.0862	0.0941
MGROS	−0.1053	0.0052	0.0951	0.0282	1.3432	0.2789	−0.1054	0.0034	0.0952	0.0288	1.3787	−0.2986	−0.0511	0.0017	0.0888	0.0217	0.5647	0.405
MPARK	−0.08	0.0042	0.0953	0.023	1.7086	0.3042	−0.078	0.002	0.0951	0.0297	0.8206	0.217	−0.0726	0.0037	0.0882	0.0285	0.1227	−0.0793
NATEN	−0.1056	0.0057	0.0954	0.0345	1.0656	0.6643	−0.1052	0.0007	0.0956	0.0485	−0.4056	0.1553	−0.1048	0.001	0.0952	0.0292	2.033	0.7236
NTGAZ	−0.11	0.0076	0.0977	0.0364	0.8233	0.6768	−0.1042	0.0006	0.0965	0.0403	0.6021	−0.1088	−0.1062	−0.0007	0.0947	0.0299	2.1591	0.0206
ODAS	−0.1054	0.0083	0.0953	0.0366	0.7043	−0.1975	−0.1052	−0.0013	0.0953	0.0397	0.0355	0.229	−0.0936	−0.0011	0.07	0.0253	0.8177	−0.3247
ORGE	−0.1054	0.0057	0.0953	0.0328	1.1851	0.5463	−0.1052	0.0034	0.0953	0.041	−0.0122	0.2262	−0.0539	0.0011	0.0946	0.0262	0.381	0.4965
PAMEL	−0.1054	0.0042	0.0952	0.0344	2.5469	−0.6694	−0.1054	−0.0035	0.0953	0.0421	0.6872	−0.0454	−0.097	−0.0003	0.0953	0.0283	1.809	0.3039
PCILT	−0.1061	0.0045	0.096	0.0339	1.3543	0.4174	−0.1056	−0.0004	0.0957	0.0502	−0.2358	0.0362	−0.1054	0.0013	0.0952	0.0354	1.2612	−0.0332
PGSUS	−0.1054	0.0067	0.095	0.0315	1.0081	0.2213	−0.1052	0.0012	0.0952	0.0308	0.6501	0.1468	−0.0626	0.0021	0.0734	0.0224	0.454	0.2579
PKENT	−0.1054	−0.0026	0.0953	0.0461	1.0746	−0.0624	−0.1054	0.0013	0.0953	0.0448	0.1823	0.2905	−0.105	0.0004	0.0953	0.0385	0.6696	0.6915
PSDTC	−0.1049	0.0017	0.0951	0.0245	2.4883	0.1978	−0.1051	0.002	0.0953	0.0426	0.069	0.3086	−0.1051	0.0017	0.0953	0.042	0.7648	0.098
RYSAS	−0.1063	0.0064	0.0955	0.0368	0.6959	0.4106	−0.1054	0.0037	0.0953	0.0413	0.2862	0.3274	−0.1051	0.0046	0.0953	0.0392	0.4166	0.5004
SANEL	−0.1053	0.0051	0.0953	0.0521	−0.3798	0.1236	−0.105	0.0004	0.0953	0.0458	−0.0276	0.1794	−0.1048	−0.0002	0.0953	0.0317	1.4653	0.6412
SANKO	−0.1049	0.0041	0.0953	0.0266	2.4172	0.537	−0.1052	0.0006	0.0953	0.0391	0.5892	0.3686	−0.1053	0.0012	0.0952	0.0309	1.5009	0.3262
SELEC	−0.1046	0.0049	0.0676	0.0247	1.126	−0.2919	−0.1054	0.0017	0.0952	0.0359	0.3766	0.1295	−0.0763	0.0009	0.0952	0.0284	0.4085	0.3469
SKYLP	−0.105	0.0009	0.0953	0.0366	1.0674	0.6092	−0.1054	0.0035	0.0953	0.0509	−0.3972	0.1061	−0.1052	0.0008	0.0953	0.0416	0.4003	0.4786
SOKM	−0.1048	0.0028	0.0947	0.0257	1.2228	−0.0354	−0.1001	0.0027	0.0946	0.0313	0.3895	−0.1458	−0.0973	−0.0011	0.0609	0.0224	1.7827	−0.4571
SONME	−0.1054	0.0004	0.0953	0.0286	2.8343	0.4779	−0.1054	0.0015	0.0952	0.0443	−0.1195	0.1434	−0.1054	0.0017	0.0953	0.0403	0.3103	0.3323
TCELL	−0.1053	0.0028	0.0948	0.0289	1.2861	0.2333	−0.1051	0.0017	0.0954	0.0322	0.4626	−0.2215	−0.0533	0.002	0.0713	0.0213	0.1825	0.0166
TEKTU	−0.1025	0.004	0.095	0.0316	0.7468	0.2466	−0.1045	−0.0009	0.0943	0.0362	0.8363	−0.2497	−0.0981	0.0022	0.0953	0.0373	0.4524	0.3217
TGSAS	−0.1053	0.0015	0.095	0.026	2.1254	−0.0291	−0.1049	0.0012	0.0953	0.0427	0.4125	0.244	−0.1048	0.0033	0.0953	0.0425	0.0372	0.4967
THYAO	−0.105	0.0074	0.0953	0.0284	1.3359	0.1066	−0.0952	0.002	0.0952	0.0314	0.4233	0.3789	−0.051	0.001	0.0641	0.0185	0.7977	0.5593
TKNSA	−0.1043	0.0056	0.0953	0.0317	1.1059	0.5772	−0.1054	0.0005	0.0953	0.039	0.0767	0.0572	−0.0709	0.001	0.0953	0.029	0.8224	0.4516
TLMAN	−0.1049	0.0028	0.0948	0.027	2.1266	0.3035	−0.1054	0.0012	0.0953	0.0377	0.5734	0.014	−0.0984	0.0006	0.0952	0.0337	0.8825	0.2851
TSPOR	−0.4597	−0.0043	0.0952	0.0477	37.9266	−4.2351	−0.1053	−0.0003	0.0953	0.0322	1.7534	0.4266	−0.0966	−0.0012	0.0948	0.0327	1.433	0.5301
TTKOM	−0.1051	0.0041	0.095	0.0307	1.5167	0.1665	−0.1054	−0.0	0.0952	0.0349	−0.0446	−0.0153	−0.0775	0.0025	0.0953	0.0251	0.9917	0.2731
TUREX	−0.1049	0.004	0.0952	0.0273	2.4937	0.8137	−0.105	−0.0001	0.0953	0.0392	0.729	0.3557	−0.1054	0.0068	0.0952	0.0327	1.5191	−0.435
TURGG	−0.1054	0.0038	0.0953	0.0346	1.3508	0.3979	−0.1053	0.0014	0.0953	0.0421	0.6031	0.1679	−0.1053	0.0009	0.0953	0.0359	1.104	0.6683
ULAS	−0.1054	0.0051	0.0953	0.0323	1.2733	0.5353	−0.1054	0.0013	0.0953	0.0489	−0.2189	0.3287	−0.1024	0.0029	0.0953	0.0457	−0.2953	0.5323
VAKKO	−0.1053	0.0074	0.0953	0.0328	1.1828	0.2476	−0.1048	0.0012	0.0953	0.0384	0.1804	0.1246	−0.0993	0.0003	0.0953	0.0284	1.6909	0.1193
YAYLA	−0.1048	0.0031	0.0951	0.0335	2.6425	0.3766	−0.1041	−0.0001	0.0953	0.0386	0.6848	0.3272	−0.1053	0.0046	0.0953	0.0541	−0.4127	0.1639
YEOTK	−0.1061	0.0064	0.0965	0.0439	0.4233	0.2325	−0.1058	0.0035	0.0955	0.0516	−0.5386	0.1859	−0.0872	0.0005	0.0953	0.0332	1.0395	0.7266
YYAPI	−0.104	0.004	0.0947	0.0345	1.1062	0.3576	−0.104	0.0006	0.0962	0.0433	−0.0053	0.318	−0.1052	0.0032	0.0956	0.0419	0.3539	0.5512
ZEDUR	−0.1055	0.0067	0.0953	0.0434	0.3503	0.0442	−0.1054	0.0006	0.0954	0.0471	−0.0958	0.27	−0.1053	−0.0013	0.0954	0.0361	1.5432	0.1661
ZOREN	−0.1045	0.0057	0.0953	0.0346	1.4526	0.3869	−0.1049	−0.0001	0.0953	0.0387	0.4081	0.3184	−0.1037	0.0002	0.0941	0.0262	2.6666	0.2469

**Table A4:** Descriptive statistics for XUTEK.

	2022						2023						2024					
Ticker	Min	Mean	Max	Std. Dev.	Kurtosis	Skewness	Min	Mean	Max	Std. Dev.	Kurtosis	Skewness	Min	Mean	Max	Std. Dev.	Kurtosis	Skewness
ALCLT	−0.1053	0.0057	0.0953	0.036	1.1785	0.0551	−0.1054	−0.0013	0.0952	0.0394	0.1522	−0.2493	−0.1048	0.0019	0.0953	0.0348	0.7991	0.2204
ARDYZ	−0.1054	0.0014	0.0955	0.0315	1.6252	−0.0119	−0.0978	0.0037	0.0953	0.0391	0.0315	0.1802	−0.1052	0.0018	0.0954	0.0357	0.3195	−0.0073
ARENA	−0.105	0.002	0.0953	0.0236	3.2312	−0.0475	−0.1021	0.0016	0.0952	0.0369	0.5064	−0.0523	−0.1054	0.0001	0.0953	0.0341	0.8939	0.109
ASELS	−0.1059	0.0042	0.0953	0.0303	1.6586	0.1901	−0.1045	0.0015	0.0948	0.034	0.3564	−0.0021	−0.0689	0.0018	0.082	0.0225	0.3415	0.1466
ATATP	−0.1056	0.0005	0.0953	0.0295	1.6687	0.4521	−0.1054	0.0052	0.0953	0.0471	−0.1357	0.1826	−0.1054	−0.0	0.0953	0.039	0.5617	0.4375
DESPC	−0.105	0.0034	0.095	0.025	3.1058	0.3136	−0.1049	0.0012	0.0953	0.038	0.3493	0.0674	−0.1047	0.0018	0.0953	0.0424	0.3836	0.3595
DGATE	−0.1049	0.0022	0.0951	0.0271	2.6135	−0.1038	−0.1053	0.0007	0.0953	0.0388	0.3415	−0.2007	−0.1053	0.0023	0.0953	0.0403	0.3879	0.1141
EDATA	−0.1054	0.0014	0.0959	0.0376	1.0794	0.228	−0.1054	0.0024	0.0953	0.042	0.2536	0.2567	−0.0856	−0.0008	0.0953	0.0338	0.2962	0.2672
ESCOM	−0.1052	0.007	0.0953	0.0344	0.5178	0.4546	−0.1016	0.0006	0.0953	0.0403	0.1931	0.0862	−0.1053	0.0011	0.0952	0.0381	0.7774	0.0319
FONET	−0.1068	0.0012	0.0961	0.0309	2.5784	0.7578	−0.103	0.0011	0.0968	0.0413	0.5331	0.1099	−0.1054	0.0039	0.0953	0.0518	−0.1314	0.1072
INDES	−0.1047	0.0045	0.08	0.0266	1.7441	−0.0944	−0.1016	0.0	0.0892	0.0343	−0.1131	0.0914	−0.0797	0.001	0.0939	0.0285	0.584	0.1803
KAREL	−0.6768	0.0029	0.0953	0.053	108.231	−8.3661	−0.1049	−0.0005	0.0951	0.0371	0.6411	0.0946	−0.0885	−0.0005	0.0797	0.0263	1.1887	0.1484
KFEIN	−0.1053	0.0019	0.0952	0.0268	2.0161	0.5211	−0.0867	0.0025	0.0953	0.0392	0.0828	0.2759	−0.1054	0.0016	0.0953	0.0426	0.3853	0.3993
KRONT	−0.6631	0.0019	0.0953	0.0531	98.2797	−7.7507	−0.1054	−0.0018	0.0953	0.0403	0.3037	−0.0277	−0.0975	0.002	0.0953	0.034	0.3842	0.1016
LINK	−0.1053	0.0029	0.0955	0.0302	2.2362	0.7878	−0.1054	0.0054	0.0953	0.0479	−0.2567	0.0867	−0.0843	0.0037	0.0953	0.0411	−0.225	0.4054
LOGO	−0.1051	0.0018	0.0767	0.0223	2.5352	−0.1703	−0.1053	0.0003	0.095	0.0326	0.4889	−0.2351	−0.0819	0.0019	0.0717	0.0296	−0.1186	−0.1935
MIATK	−0.1054	0.0048	0.0958	0.0372	1.3106	0.487	−0.1055	0.0054	0.0956	0.0525	−0.5228	0.1161	−0.1033	0.0007	0.0953	0.0394	0.5074	0.5238
MOBTL	−0.1022	0.0038	0.0946	0.0311	1.0094	0.3376	−0.1045	−0.0006	0.095	0.0359	0.9626	0.0238	−0.1048	0.0006	0.0951	0.0336	1.4219	0.3114
MTRKS	−0.1055	0.0032	0.0954	0.035	2.7227	−0.6161	−0.1052	0.0004	0.0953	0.0353	1.0621	0.0187	−0.1054	0.0016	0.095	0.0368	0.2651	−0.005
NETAS	−0.105	0.0035	0.0953	0.0299	2.0508	0.044	−0.1051	−0.0001	0.0953	0.0442	−0.2673	0.055	−0.1054	0.0019	0.0953	0.0374	0.7613	0.2699
PENTA	−0.1053	0.0005	0.0954	0.0409	0.9268	0.4574	−0.1054	−0.0044	0.0953	0.0428	0.3636	0.1104	−0.0939	−0.0007	0.0953	0.0269	2.7586	0.605
PKART	−0.1049	0.002	0.0953	0.0308	1.9578	0.4431	−0.1054	0.0002	0.0953	0.0413	0.1914	0.0794	−0.1052	0.0032	0.0953	0.0444	0.2116	0.5098
SMART	−0.1047	0.0029	0.0953	0.0323	1.9421	0.768	−0.1052	0.0036	0.0953	0.0518	−0.5587	0.1265	−0.1049	−0.0	0.0953	0.0385	0.9699	0.7756
VBTYZ	−0.1054	0.0039	0.0959	0.0319	2.5431	0.3589	−0.1049	0.003	0.0957	0.041	0.5431	0.262	−0.1051	−0.0002	0.0952	0.0351	1.2704	0.6536

**Table A5:** Descriptive statistics for XBANK.

	2022						2023						2024					
Ticker	Min	Mean	Max	Std. Dev.	Kurtosis	Skewness	Min	Mean	Max	Std. Dev.	Kurtosis	Skewness	Min	Mean	Max	Std. Dev.	Kurtosis	Skewness
AKBNK	−0.1052	0.0039	0.0953	0.0334	1.9246	−0.0302	−0.1048	0.0029	0.0955	0.0347	0.9681	0.1607	−0.0869	0.0023	0.0947	0.0286	0.3872	0.1474
ALBRK	−0.1007	0.0034	0.0953	0.0318	1.9447	0.3915	−0.0999	0.0005	0.0953	0.0282	2.3027	0.0757	−0.0513	0.002	0.072	0.0235	0.4423	0.2644
GARAN	−0.1051	0.0037	0.0953	0.0321	2.4488	0.079	−0.1055	0.003	0.0953	0.0356	0.8114	0.0896	−0.0621	0.0033	0.0786	0.0244	0.108	0.2185
HALKB	−0.1052	0.0042	0.0953	0.0388	1.4336	0.1389	−0.1047	−0.0004	0.0953	0.0326	1.1334	0.1325	−0.0839	0.0012	0.095	0.0267	1.8555	0.4468
ICBCT	−0.1049	0.003	0.0953	0.032	2.6558	0.1537	−0.1054	0.0009	0.0953	0.036	0.7748	0.3547	−0.0856	−0.0001	0.095	0.0254	1.2522	0.2841
ISCTR	−0.1053	0.0054	0.0954	0.0356	1.5001	−0.0767	−0.1048	0.0027	0.0952	0.0358	0.9271	0.1375	−0.0668	0.0015	0.0875	0.0249	0.2901	0.1567
SKBNK	−0.2133	0.0033	0.0957	0.0427	3.1582	−0.6794	−0.1055	0.0015	0.0952	0.0392	0.9152	−0.0687	−0.0904	0.0014	0.0934	0.0263	1.3366	0.1884
TSKB	−0.1054	0.0043	0.0953	0.0419	0.9086	−0.2107	−0.1051	0.0016	0.0953	0.0365	0.6322	−0.0124	−0.0843	0.0025	0.0951	0.0247	0.9922	−0.0035
VAKBN	−0.1052	0.0046	0.0953	0.0378	1.6845	0.136	−0.1043	0.0003	0.0951	0.0335	0.615	0.055	−0.0663	0.0023	0.0953	0.0269	0.7171	0.1925
YKBNK	−0.1049	0.005	0.0952	0.0346	1.0973	−0.1263	−0.1051	0.0024	0.095	0.034	1.5978	−0.0413	−0.0704	0.0018	0.0953	0.0279	0.5735	0.2798

## Appendix B. Sensitivity Analysis

The sensitivity analysis is designed to evaluate how different rolling window lengths affect the computation of sample entropy—a measure of the complexity in the log-return series of company prices. Using window lengths of 10, 15, and 30 days across various sectors, the analysis investigates the robustness of the rolling metric and its ability to capture the underlying dynamics of the data. Statistical testing via the Kolmogorov–Smirnov (KS) test and bootstrap resampling methods for estimating 95% confidence intervals further reinforces the evaluation by quantifying the significance of differences between these window lengths and by demonstrating the reliability of the computed entropy values.

In this analysis, the primary parameters include the candidate window lengths of 10, 15, and 30 days, an embedding dimension set to 2 for the sample entropy calculation, and the Chebyshev distance metric. The KS test compares the distributions of sample entropy values obtained with each window length, while bootstrapping—with 1000 resamples—is employed to construct 95% confidence intervals for the mean sample entropy. These settings ensure that the analysis captures both the variability and the statistical robustness of the entropy estimates across different time scales.

The analysis results are presented in Table A6 and Table A7.

**Table A6:** Kolmogorov–Smirnov Test Results for Rolling Window Sensitivity Analysis by Sector.

Sector	Window Pair	KS Statistic	*p*-Value
XBANK	10-day vs. 15-day	0.9000	0.0002
XBANK	15-day vs. 30-day	1.0000	0.0000
XELKT	10-day vs. 15-day	0.8889	0.0000
XELKT	15-day vs. 30-day	1.0000	0.0000
XUHIZ	10-day vs. 15-day	0.9111	0.0000
XUHIZ	15-day vs. 30-day	1.0000	0.0000
XUSIN	10-day vs. 15-day	0.9758	0.0000
XUSIN	15-day vs. 30-day	1.0000	0.0000
XUTEK	10-day vs. 15-day	0.9200	0.0000
XUTEK	15-day vs. 30-day	1.0000	0.0000

**Table A7:** Aggregated Sample Entropy and Bootstrap Confidence Intervals by Sector and Window Length.

Sector	Window Length (Days)	Mean Sample Entropy	*n*	95% CI Lower	95% CI Upper
XBANK	10	0.0403	10	0.0240	0.0596
XBANK	15	0.1973	10	0.1464	0.2601
XBANK	30	1.4687	10	1.4031	1.5240
XELKT	10	0.0408	18	0.0304	0.0506
XELKT	15	0.2439	18	0.1973	0.2915
XELKT	30	1.5318	18	1.4978	1.5660
XUHIZ	10	0.0424	90	0.0377	0.0481
XUHIZ	15	0.2422	90	0.2217	0.2609
XUHIZ	30	1.5325	90	1.5156	1.5483
XUSIN	10	0.0347	165	0.0319	0.0374
XUSIN	15	0.2135	165	0.2020	0.2256
XUSIN	30	1.5315	165	1.5179	1.5458
XUTEK	10	0.0370	25	0.0284	0.0493
XUTEK	15	0.1988	25	0.1666	0.2397
XUTEK	30	1.5117	25	1.4781	1.5436

The results reveal a clear pattern across all sectors. The 10-day window consistently produces very low average sample entropy values (for example, 0.0403 in XBANK, 0.0408 in XELKT, 0.0424 in XUHIZ, 0.0347 in XUSIN, and 0.0370 in XUTEK), while the 30-day window yields much higher values (1.4687 in XBANK, 1.5318 in XELKT, 1.5325 in XUHIZ, 1.5315 in XUSIN, and 1.5117 in XUTEK). The 15-day window falls between these extremes, with mean entropy values of 0.1973, 0.2439, 0.2422, 0.2135, and 0.1988 for XBANK, XELKT, XUHIZ, XUSIN, and XUTEK, respectively. The KS tests conducted between the 10- and 15-day windows and between the 15- and 30-day windows yield very high KS statistics and extremely low *p*-values (often 0.0000 or 0.0002), indicating that the entropy distributions differ significantly between these window lengths. Additionally, the bootstrap confidence intervals for the 15-day window are relatively narrow—for instance, in the XBANK sector, the mean of 0.1973 has a 95% confidence interval ranging from 0.1464 to 0.2601—underscoring the reliability of the estimate at this window size.

Choosing a 15-day window over the 10- and 30-day alternatives is justified by the balance it strikes between capturing sufficient complexity and avoiding excessive noise. The 10-day window, being very short, underestimates the system’s complexity due to its limited data points, while the 30-day window overestimates complexity by incorporating too much variability and potentially smoothing out critical short-term dynamics. In contrast, the 15-day window provides a more moderate and stable estimate of sample entropy that reflects meaningful short-term variations without being overly influenced by noise. This balance, along with the significant statistical differences observed between the entropy distributions and the robust confidence intervals, makes the 15-day window a suitable choice for capturing the dynamic evolution of the market.

## References

[1] Tiniç M., Savaser T. (2020). Political turmoil and the impact of foreign orders on equity prices. J. Int. Financ. Mark. Inst. Money.

[2] Yilmaz M.K., Aksoy M., Tatoglu E. (2020). Does the stock market value inclusion in a sustainability index? Evidence from Borsa Istanbul. Sustainability.

[3] Balcı M.A., Batrancea L.M., Akgüller Ö., Gaban L., Rus M.I., Tulai H. (2022). Fractality of Borsa Istanbul during the COVID-19 pandemic. Mathematics.

[4] Kirikkaleli D., Kayar E.Ü. (2023). The effect of economic, financial and political stabilities on the banking sector: Cases of six balkan countries. Sustainability.

[5] Ekinci C., Ersan O. (2024). Impact of the COVID-19 Market Turmoil on Investor Behavior: A Panel VAR Study of Bank Stocks in Borsa Istanbul. Int. J. Financ. Stud..

[6] Batrancea L.M., Balcı M.A., Akgüller Ö., Nichita A. (2024). The impact of social media discourse on financial performance of e-commerce companies listed on Borsa Istanbul. Humanit. Soc. Sci. Commun..

[7] Blume M.E., Friend I. (1973). A new look at the capital asset pricing model. J. Financ..

[8] Elbannan M.A. (2015). The capital asset pricing model: An overview of the theory. Int. J. Econ. Financ..

[9] Huberman G. (1982). A simple approach to arbitrage pricing theory. J. Econ. Theory.

[10] Roll R., Ross S.A. (1980). An empirical investigation of the arbitrage pricing theory. J. Financ..

[11] Zerbib O.D. (2022). A sustainable capital asset pricing model (S-CAPM): Evidence from environmental integration and sin stock exclusion. Rev. Financ..

[12] Gu S., Kelly B., Xiu D. (2021). Autoencoder asset pricing models. J. Econom..

[13] Hoque M.E., Zaidi M.A.S. (2020). Global and country-specific geopolitical risk uncertainty and stock return of fragile emerging economies. Borsa Istanb. Rev..

[14] Ayub U., Kausar S., Noreen U., Zakaria M., Jadoon I.A. (2020). Downside risk-based six-factor capital asset pricing model (CAPM): A new paradigm in asset pricing. Sustainability.

[15] Peng W., Wen M., Jiang X., Li Y., Chen T., Zheng B. (2024). Global motion filtered nonlinear mutual information analysis: Enhancing dynamic portfolio strategies. PLoS ONE.

[16] Hammad M., Chelloug S.A., Alayed W., El-Latif A.A.A. (2023). Optimizing Multimodal Scene Recognition through Mutual Information-Based Feature Selection in Deep Learning Models. Appl. Sci..

[17] Zhao K., Dong G., Bian D. (2023). Detection of illegal transactions of cryptocurrency based on mutual information. Electronics.

[18] Hope O.K., Rao P., Xu Y., Yue H. (2023). Information sharing between mutual funds and auditors. J. Bus. Financ. Account..

[19] Goldstein I. (2023). Information in financial markets and its real effects. Rev. Financ..

[20] Massara G.P., Di Matteo T., Aste T. (2016). Network filtering for big data: Triangulated maximally filtered graph. J. Complex Netw..

[21] Millington T. (2022). An investigation into the effects and effectiveness of correlation network filtration methods with financial returns. PLoS ONE.

[22] Balcı M.A., Batrancea L.M., Akgüller Ö., Nichita A. (2022). Coarse graining on financial correlation networks. Mathematics.

[23] Eggels A., Crommelin D. (2019). Quantifying data dependencies with Rényi mutual information and minimum spanning trees. Entropy.

[24] Stam C., Tewarie P., Van Dellen E., Van Straaten E., Hillebrand A., Van Mieghem P. (2014). The trees and the forest: Characterization of complex brain networks with minimum spanning trees. Int. J. Psychophysiol..

[25] Zhao X., Shang P., Huang J. (2017). Mutual-information matrix analysis for nonlinear interactions of multivariate time series. Nonlinear Dyn..

[26] Contreras-Reyes J.E. (2021). Mutual information matrix based on asymmetric Shannon entropy for nonlinear interactions of time series. Nonlinear Dyn..

[27] Tumminello M., Aste T., Di Matteo T., Mantegna R.N. (2005). A tool for filtering information in complex systems. Proc. Natl. Acad. Sci. USA.

[28] Forman R. (2003). Bochner’s method for cell complexes and combinatorial Ricci curvature. Discret. Comput. Geom..

[29] Samal A., Sreejith R., Gu J., Liu S., Saucan E., Jost J. (2018). Comparative analysis of two discretizations of Ricci curvature for complex networks. Sci. Rep..

[30] Saucan E., Sreejith R., Vivek-Ananth R., Jost J., Samal A. (2019). Discrete Ricci curvatures for directed networks. Chaos Solitons Fractals.

[31] Fesser L., de Haro Ivánez S.S., Devriendt K., Weber M., Lambiotte R. (2024). Augmentations of Forman’s Ricci curvature and their applications in community detection. J. Phys. Complex..

[32] Murgas K.A., Saucan E., Sandhu R. (2022). Hypergraph geometry reflects higher-order dynamics in protein interaction networks. Sci. Rep..

[33] Chatterjee T., Albert R., Thapliyal S., Azarhooshang N., DasGupta B. (2021). Detecting network anomalies using Forman–Ricci curvature and a case study for human brain networks. Sci. Rep..

[34] Gunes S., Karaalp H.S. (2012). Exports and economic growth: A sectoral analysis for Turkey. Int. J. Econ. Financ. Stud..

[35] Konak F., Güner E.N. (2016). The impact of working capital management on firm performance: An empirical evidence from the BIST SME industrial index. Int. J. Trade, Econ. Financ..

[36] Güven F. (2024). Reshaping the Industrial Policy Framework of Turkiye: Integrating Exports at the Core of AI-Driven Development. Ind. Policy.

[37] Huseynli N. (2022). The relationship between consumer confidence index and BIST 50 index. J. East. Eur. Cent. Asian Res. (JEECAR).

[38] Kurtaran A.T., Günay B., Kurtaran A. (2021). A Model Suggestion for Determining the Values of Firms the Energy Sector: An Application in BIST Electricity Index. Int. J. Energy Econ. Policy.

[39] Huseynli N. (2023). BIST tourism and BIST electricity index relationship. Int. J. Energy Econ. Policy.

[40] Gökhan Ö., Özen A.K. (2017). Ownership structure in BIST-Capital structure relation Granger causality test a comparative application between BIST industrial index and service index. J. Econ. Bibliogr..

[41] Bouaziz Z. (2016). The impact of R&D expenses on firm performance: Empirical witness from the Bist Technology Index. J. Bus. Theory Pract..

[42] Kocaarslan B. (2020). Volatility Transmission Between US Economic Policy Uncertainty and BIST (Borsa Istanbul) Major Sector Indices. Bus. Manag. Stud. Int. J..

[43] Tekin B. (2021). Financial Ratios Affecting Systematic Risk in Joint-Stock Companies: BIST Technology (XUTEK) Industry Companies Case in Turkey. Financ. Stud..

[44] Altuntaş D., Ersoy E. (2022). The Causal Relationship between CDS Premiums and BIST 30 and BIST Banks Index. J. Econ. Financ. Res..

[45] Saydar O.O., Bedir C. (2021). Value investing analysis of banking sector on BIST-100. J. Econ. Financ. Account..

[46] Armagan I.U. (2023). Price prediction of the Borsa Istanbul banks index with traditional methods and artificial neural networks. Borsa Istanb. Rev..

[47] Hailu S.M., Vural G. (2020). Testing the weak form market efficiency of Borsa Istanbul: An empirical evidence from Turkish banking sector stocks. J. Econ. Financ. Account..

[48] Shannon C.E. (1948). A mathematical theory of communication. Bell Syst. Tech. J..

[49] Kreer J. (1957). A question of terminology. IRE Trans. Inf. Theory.

[50] Granger C.W. (1969). Investigating causal relations by econometric models and cross-spectral methods. Econom. J. Econom. Soc..

[51] Schreiber T. (2000). Measuring information transfer. Phys. Rev. Lett..

